# A Screen Using iPSC-Derived Hepatocytes Reveals NAD^+^ as a Potential Treatment for mtDNA Depletion Syndrome

**DOI:** 10.1016/j.celrep.2018.10.036

**Published:** 2018-11-06

**Authors:** Ran Jing, James L. Corbett, Jun Cai, Gyda C. Beeson, Craig C. Beeson, Sherine S. Chan, David P. Dimmock, Lynn Lazcares, Aron M. Geurts, John J. Lemasters, Stephen A. Duncan

**Affiliations:** 1 Department of Regenerative Medicine and Cell Biology, Medical University of South Carolina, 173 Ashley Avenue, Charleston, SC 29425, USA; 2 Department of Drug Discovery and Biomedical Sciences, Medical University of South Carolina, 173 Ashley Avenue, Charleston, SC 29425, USA; 3 Human Molecular Genetics Center and Division of Genetics, Department of Pediatrics, Medical College of Wisconsin, 8701 Watertown Plank Road, Milwaukee, WI 53226, USA; 4 Rady Children’s Institute for Genomic Medicine, 3020 Children’s Way, San Diego, CA 92123, USA; 5 Division of Pediatric Pathology, Department of Pathology and Laboratory Medicine, Medical College of Wisconsin, 8701 Watertown Plank Road, Milwaukee, WI 53226, USA; 6 Department of Physiology, Medical College of Wisconsin, 8701 Watertown Plank Road, Milwaukee, WI 53226, USA; 7 Center for Cell Death, Injury and Regeneration, Department of Drug Discovery and Biomedical Sciences, Medical University of South Carolina, Charleston, SC 29425, USA; 8 Hollings Cancer Center, Medical University of South Carolina, 86 Jonathan Lucas Street, Charleston, SC 29425, USA; 9 Lead Contact

## Abstract

Patients with mtDNA depletion syndrome 3 (MTDPS3) often die as children from liver failure caused by severe reduction in mtDNA content. The identification of treatments has been impeded by an inability to culture and manipulate MTDPS3 primary hepatocytes. Here we generated DGUOK-deficient hepatocyte-like cells using induced pluripotent stem cells (iPSCs) and used them to identify drugs that could improve mitochondrial ATP production and mitochondrial function. Nicotinamide adenine dinucleotide (NAD) was found to improve mitochondrial function in DGUOK-deficient hepatocyte-like cells by activating the peroxisome proliferator-activated receptor gamma coactivator 1-alpha (PGC1 α). NAD treatment also improved ATP production in MTDPS3-null rats and in hepatocyte-like cells that were deficient in ribonucleoside-diphosphate reductase subunit M2B (RRM2B), suggesting that it could be broadly effective. Our studies reveal that DGUOK-deficient iPSC-derived hepatocytes recapitulate the pathophysiology of MTDPS3 in culture and can be used to identify therapeutics for mtDNA depletion syndromes.

## In Brief

Jing et al. show that a drug screen using iPSC-derived hepatocytes that harbor a mutation in the DGUOK gene leads to the identification of potential treatments for mtDNA depletion syndromes. NAD, a bioactive form of niacin, increases ATP production and mitochondrial function in DGUOK-deficient hepatocytes and rats.

## Graphical Abstract

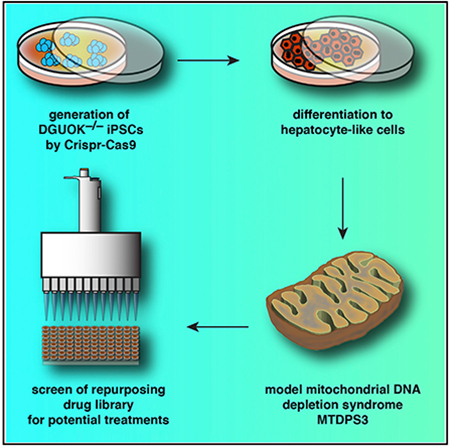

## INTRODUCTION

The primary function of mitochondria is to provide energy for a variety of biological processes through oxidative phosphorylation. Unlike other cellular organelles whose function is dependent solely on the transcription of nuclear DNA, mitochondria maintain several copies of their own genome (mtDNA). The mtDNA is essential for ATP production through oxidative phosphorylation because it encodes a subset of proteins that form the electron transport chain (ETC) complexes. mtDNA depletion syndromes (MTDPSs) are a group of genetic disorders characterized by depletion of mtDNA and reduced ATP synthesis, leading to disease in multiple tissues. One of the leading causes of death in MTDPS patients is liver dysfunction. The mtDNA depletion results from mutations in genes that encode enzymes that are required to maintain the mitochondrial dNTP pool (Mandel et al., 2001) or regulate mtDNA replication (Van Goethem et al., 2001; Sarzi et al., 2007). Among these diseases, deoxyguanosine kinase (DGUOK) deficiency is the most common cause of hepatic mtDNA depletion syndrome and accounts for approximately 15%–20% of all MTDPS cases (Sezer et al., 2015).

*DGUOK* is a nuclear gene that encodes a mitochondrial kinase responsible for the phosphorylation of purine deoxyribonucleosides. DGUOK deficiency prevents the production of deoxyadenosine monophosphate (dAMP) and deoxyguanosine monophosphate (dGMP) (Gower et al., 1979). The lack of available nucleotides within the mitochondria results in a reduction of mtDNA copy number in DGUOK-deficient hepatocytes (Dimmock et al., 2008b). Depending on the type of mutations, DGUOK-related MTDPS, also called mtDNA depletion syndrome 3 (MTDPS3), can cause neonatal hepatic disorders or multisystem diseases (Dimmock et al., 2008a, 2008b). Despite the heterogeneity of clinical phenotypes, most MTDPS3 patients suffer from hypoglycemia, lactic acidosis, and progressive liver disease and commonly die from liver failure in infancy or early childhood (Mandel et al., 2001; Salviati et al., 2002; Mancuso et al., 2005; Dimmock et al., 2008b). No cure is available for MTDPS3, and all current treatments are palliative. Though patients with isolated liver disease can benefit from liver transplantation, the survival rate is low, especially when neurological manifestations are present (Dimmock et al., 2008a). In reality, the variability in outcome associated with liver transplantation in MTDPS3 patients coupled with a shortage of available liver donors precludes transplantation as a viable treatment, so there is a clear need for alternatives.

The identification of treatments for MTDPS3 has been impeded by the scarcity of liver samples from patients with severe DGUOK deficiencies. Recently, human induced pluripotent stem cells (iPSCs) combined with gene editing have offered an opportunity to model even the rarest of rare diseases in culture without the need to access patients directly. In the present study, we generated DGUOK loss-of-function iPSCs using CRISPR/Cas9 and differentiated the *DGUOK*^−/−^ iPSCs into hepatocyte-like cells that recapitulate the mitochondrial dysfunction that is associated with the livers of MTDPS3 patients. We next used the DGUOK-deficient hepatocyte-like cells as a platform to identify drugs that could improve mitochondrial function and ATP production in both DGUOK-deficient iPSC-derived hepatocytes and in the livers of *DGUOK*^−/−^ rats. We propose that such drugs have therapeutic potential for the treatment of various mtDNA depletion syndromes.

## RESULTS

### Differentiation of iPSCs to Hepatocyte-like Cells Is Accompanied by a Switch from Glycolysis to Mitochondrial Respiration

Multiple protocols have been developed to differentiate iPSCs to hepatocyte-like cells, and the resulting cells express hepatic markers and exhibit many functions associated with hepatocytes (Si-Tayeb et al., 2010; Touboul et al., 2010). Researchers have previously examined mitochondrial maturation during the formation of iPSC-derived hepatocytes and confirmed an increase of mtDNA copy number, mitochondrial gene expression, cristae structure, and oxygen consumption in iPSC-derived hepatocytes compared with undifferentiated cells (Yu et al., 2012; Hopkinson et al., 2017). The high metabolic activity of hepatocytes makes them especially dependent on efficient mitochondrial function to provide ATP, while iPSCs rely largely on glycolysis as the source of energy production (Folmes et al., 2011; Zheng et al., 2016).

**Figure 1.**
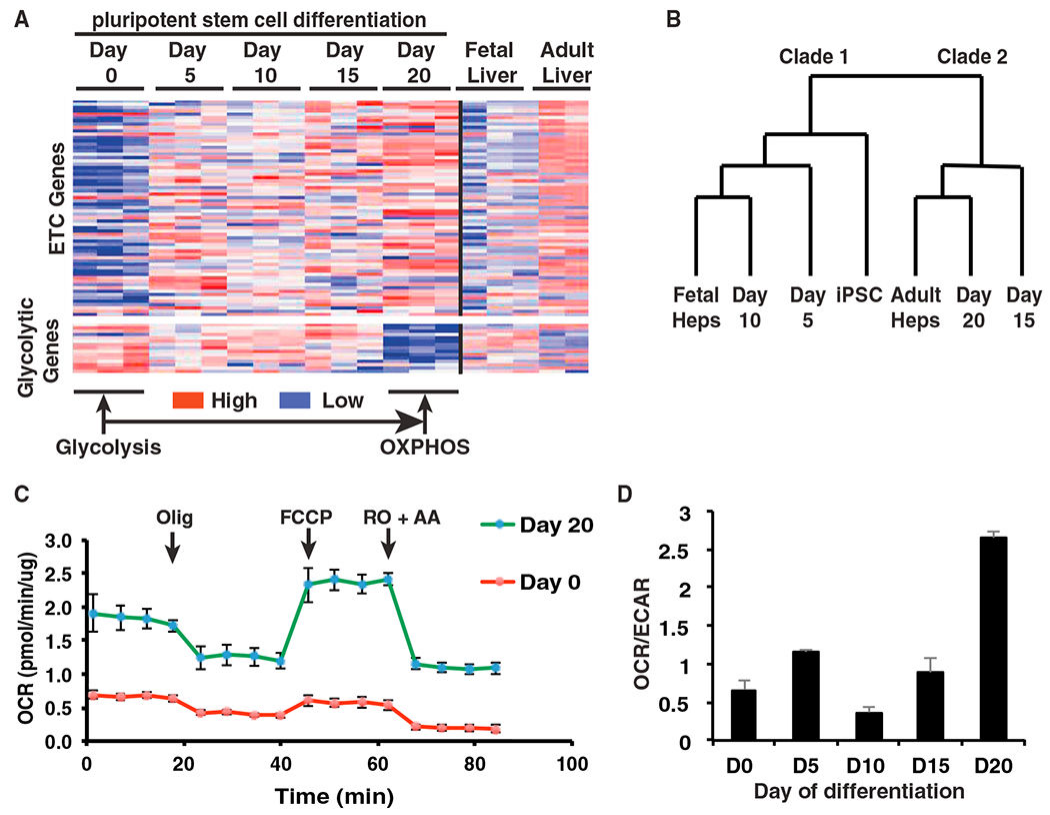
Characterization of Glucose Metabolic Pathways during Human iPSC Differentiation to Hepatocyte-like Cells (A) Heatmap showing relative abundance of 82 mRNAs encoding proteins with roles in glucose metabolism (67 electron transport chain genes and 15 glycolytic genes) (n = 3). (B) Dendrogram representing hierarchical cluster analyses of expression data. (C) OCR of day 0 iPSCs (red) and day 20 hepatocyte-like cells (green) differentiated from wild-type iPSCs, determined by Seahorse assay (n = 3, mean ± SEM). Olig, oligomycin (1 μM); FCCP, carbonyl cyanide-p-trifluoromethoxyphenylhydrazone (1 μM); RO, rotenone (2 μM); AA, antimycin A (1 μM). (D) Bar graph showing the ratio of OCR to ECAR during iPSC differentiation as determined by Seahorse assay (n = 3 biological replicates, mean ± SEM).

For iPSC-derived hepatocytes to be an effective model of mitochondrial dysfunction associated with MTDPS3 requires control cells to display active mitochondrial function. We therefore further assessed mitochondrial respiration by comparing metabolic gene expression among iPSCs, iPSC-derived hepatic cells, fetal livers, and adult livers using transcriptome analyses. We first examined the mRNA levels from 15 genes that were defined by the Gene Ontology Consortium (http://www.geneontology.org) as encoding proteins with roles in glycolysis and 67 genes that encoded proteins involved in electron transport chain and mitochondrial respiration (Figure 1A). As expected, expression of electron transport chain genes was low in human fetal livers (20–38 weeks of gestation), which rely on glycolysis for ATP production, but high in adult livers that primarily use mitochondrial respiration (Sadava et al., 1992). Both iPSCs and cells at early stages of differentiation primarily expressed glycolytic genes. However, as the iPSC-derived hepatocytes matured, between days 15 and 20 of differentiation, glycolytic gene expression was reduced, and the expression of electron transport chain genes increased. Hierarchical cluster analyses of the expression data revealed that the undifferentiated iPSCs formed a clade containing hepatic lineages from the earliest stages of differentiation as well as fetal hepatocytes. In contrast, day 15 and 20 differentiated cells segregated with adult hepatocytes, indicating that they had adopted a metabolic gene expression profile comparable with that of the adult liver (Figure 1B). To confirm the transcriptome analysis, we next performed functional assays to determine the mitochondrial activity in iPSC-derived hepatocytes. We first confirmed that iPSCs were capable of differentiating into hepatocyte-like cells using plates that were compatible with Seahorse analyses. Immunostaining and ELISA were performed to determine the expression of characteristic hepatocyte proteins HNF4A and APOB (Figures S1A and S1B). We next measured the oxygen consumption rate (OCR) in undifferentiated iPSCs and hepatocyte-like cells (day 20). Undifferentiated iPSCs displayed a low level of oxidative phosphorylation (OXPHOS) activity and low respiration capacity and responded poorly to electron transport chain inhibitors and uncouplers. In contrast, iPSC-derived hepatocytes were metabolically active, with relatively high levels of oxidative phosphorylation activity and basal respiration capacity and responded robustly to the compounds targeting the electron transport chain complexes (Figure 1C). We next measured the extracellular acidification rate (ECAR) as a reflection of glycolysis, and the ratio of OCR to ECAR was calculated as an indication of cellular metabolic preference (Zhang et al., 2012). An increase of the OCR-to-ECAR ratio confirmed the switch from glycolysis to oxidative respiration during differentiation (Figure 1D). On the basis of these data, we conclude that the differentiation of iPSCs into hepatocyte-like cells recapitulates the change in metabolic phenotype observed during hepatogenesis and that the day 20 hepatocyte-like cells primarily rely on mitochondrial respiration for energy production.

### Generation of *DGUOK*-Mutant iPSC-Derived Hepatocytes

**Figure 2.**
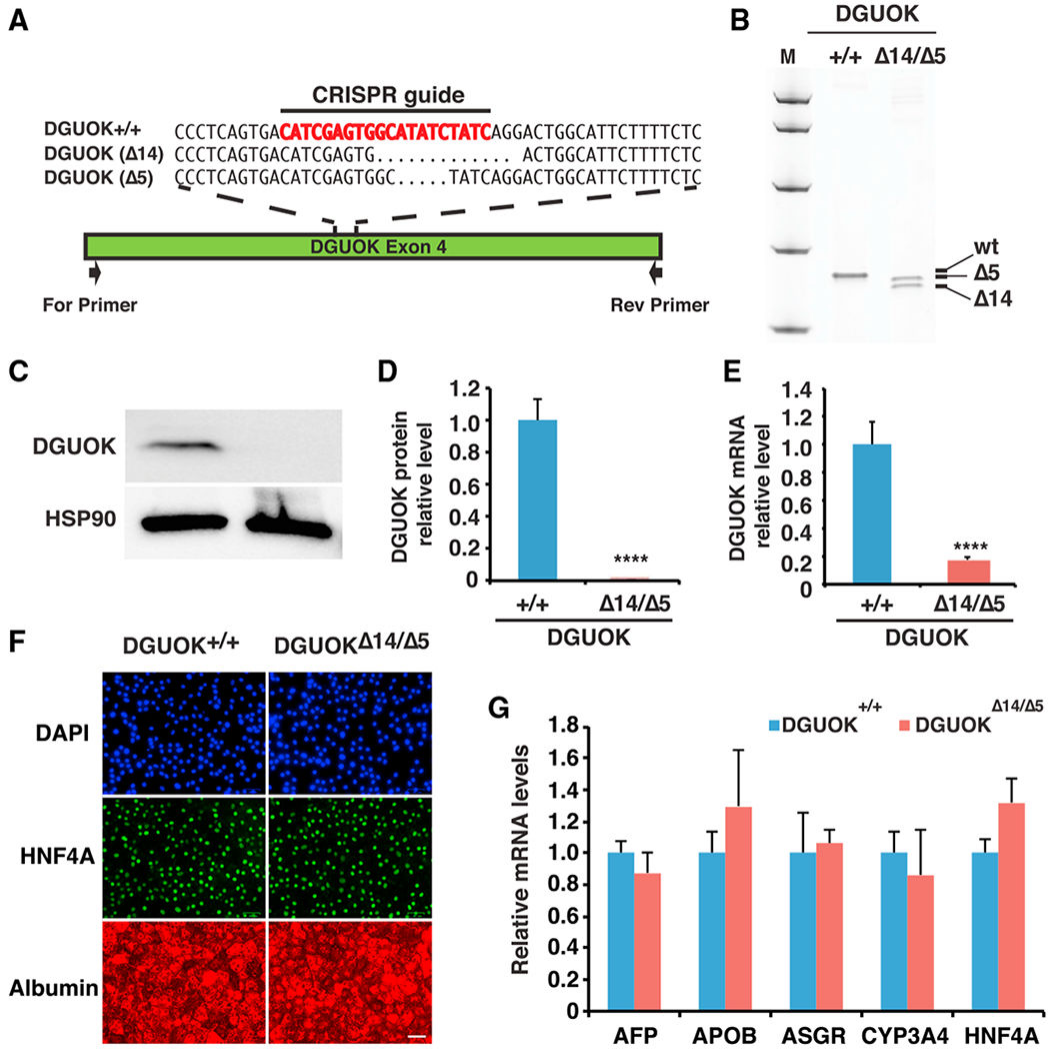
Generation of DGUOK^Δ14/Δ5^ iPSC-Derived Hepatocyte-like Cells (A) Schematic illustration of DGUOK exon 4 showing the CRISPR/Cas9 guide (red) and nucleotide sequences of DGUOK wild-type (DGUOK^+/+^) and mutant alleles (DGUOK^Δ14/Δ5^). Black arrow showing relative position of PCR primers used to identify indels. (B) Image of the polyacrylamide gel showing DGUOK exon 4 amplicons from control iPSCs (+/+) and iPSCs harboring compound heterozygous deletions of 14 and 5 bp within DGUOK exon 4 (Δ14/Δ5). (C) Immunoblot to detect DGUOK in either control (+/+) or DGUOK^Δ14/Δ5^ hepatocytes. HSP90 was used as a loading control. (D) Quantification of DGUOK protein by densitometry of immunoblots. DGUOK protein was normalized to total protein (n = 3 biological replicates, mean ± SEM, ****p ≤ 0.0001). (E) Bar graph showing relative steady-state level of DGUOK mRNA in DGUOK^+/+^ and DGUOK^Δ14/Δ5^ iPSCs (n = 3 biological replicates, mean ± SEM, ****p ≤ 0.0001). (F) Representative fluorescent images of DAPI (blue), HNF4A (green), and albumin (red) staining on day 20 wild-type and DGUOK^Δ14/Δ5^ hepatocyte-like cells. Scale bar, 50 μm. (G) Bar graph showing relative expression levels of hepatic genes in DGUOK^+/+^ and DGUOK^Δ14/Δ5^ hepatocytes at day 20 of differentiation (n = 3 biological replicates, mean ± SEM).

MTDPS3 is a rare disease, which results in extremely sick infants with a limited lifespan. As a consequence, obtaining liver biopsies is challenging. In contrast, introducing mutations into an existing well-characterized iPSC line is relatively simple and ensures that genomic variations present in patients, which are independent of the DGUOK locus but could potentially affect differentiation efficiencies, will be excluded. To determine whether an iPSC-hepatocyte model could mirror the loss of DGUOK found in MTDPS3 livers, we first generated DGUOK-deficient iPSCs using CRISPR/Cas9 (Figure 2A). We chose to mutate Exon4 on the basis of studies of patients with a similar deletion. We generated a compound heterozygote iPSC line (*DGUOK*^Δ14/Δ5^) that contained frameshift deletions of 14 and 5 bp (p.[Trp166Ter];[His167LeufsTer213]) (Figures 2A and 2B), which were confirmed by DNA sequencing. Wild-type and *DGUOK*^Δ14/Δ5^ iPSCs were then differentiated into hepatocytes using our previously described protocol (Mallanna and Duncan, 2013; Si-Tayeb et al., 2010). Immunoblot analysis found DGUOK protein to be undetectable in *DGUOK*^Δ14/Δ5^ iPSC-derived hepatocytes (Figures 2C and 2D). Furthermore, the mRNA levels of DGUOK were reduced by 5-fold in the *DGUOK*^Δ14/Δ5^ hepatocytes, which was likely the result of nonsense-mediated decay of the transcript (Figure 2E). Immunostaining (Figure 2F) and qRT-PCR (Figure 2G) showed that day 20 *DGUOK*^Δ14/Δ5^ cells expressed hepatic markers at the same level as control cells, confirming that the *DGUOK*^Δ14/Δ5^ iPSCs can be efficiently differentiated into hepatocyte-like cells.

### DGUOK-Deficient iPSC-Derived Hepatocyte-like Cells Recapitulate the Pathophysiology of MTDPS3

**Figure 3.**
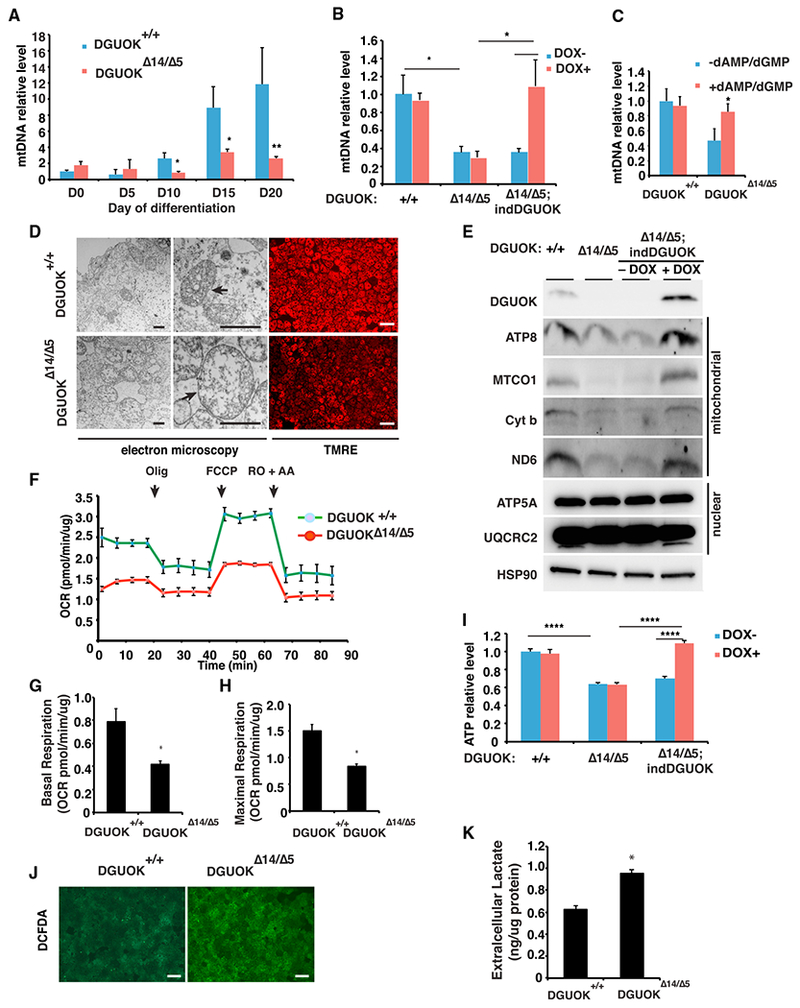
Analysis of DGUOK-Deficient iPSC-Derived Hepatocyte-like Cells (A) Bar graph showing mtDNA content during the differentiation of wild-type (blue) and DGUOK^Δ14/Δ5^ (red) iPSCs (n = 8 biological replicates, mean ± SEM, *p ≤ 0.05, **p ≤ 0.001). (B) Bar graph showing mtDNA content in iPSC-derived hepatocyte-like cells from wild-type, DGUOK^Δ14/Δ5^, and DGUOK^Δ14/Δ5;;indDGUOK^ iPSCs in the absence (blue) or presence (red) of Dox (10 ng/mL) (n = 8 biological replicates, mean ± SEM, *p ≤ 0.05). (C) Bar graph showing mtDNA content in day 20 hepatocytes from wild-type and DGUOK^Δ14/Δ5^ iPSCs with (blue) or without (red) treatment with dAMP and dGMP (n = 16, mean ± SEM, *p ≤ 0.05). (D) Micrographs showing electron microscopy (left and middle) and TMRE staining (right) of day 20 DGUOK^+/+^ and DGUOK^Δ14/Δ5^ hepatocytes. Arrows, mitochondria. Black scale bar, 800 nm; white scale bar, 100 μm. (E) Immunoblot showing levels of electron transport chain proteins encoded by mitochondrial or nuclear genomes in day 20 wild-type (+/+), DGUOK^Δ14/Δ5^, and DGUOK^Δ14/Δ5;indDGUOK^ hepatocytes in the presence (+) or absence (−) of Dox (10 ng/mL). HSP90 was used as a loading control. (F) Graph showing oxygen consumption rate (OCR) of control (green) and DGUOK^Δ14/Δ5^ (red) hepatocytes at day 20 of differentiation as determined by Seahorse assay (n = 3, mean ± SEM). (G) Basal respiration in DGUOK^+/+^ and DGUOK^Δ14/Δ5^ iPSC-derived hepatocyte-like cells (mean ± SEM, n = 3, *p ≤ 0.05). (H) Maximal respiration in DGUOK^+/+^ and DGUOK^Δ14/Δ5^ iPSC-derived hepatocyte-like cells (mean ± SEM, n = 3, *p ≤ 0.05). (I) Bar graph showing intracellular ATP levels in day 20 DGUOK^+/+^, DGUOK^Δ14/Δ5^, and DGUOK^Δ14/Δ5;;indDGUOK^ hepatocytes in the presence (red) or absence (blue) of Dox (10 ng/mL) (n = 8, mean ± SEM, ****p ≤ 0.0001). (J) ROS levels measured by DCFDA staining in day 20 DGUOK^+/+^ and DGUOK^Δ14/Δ5^ hepatocytes (scale bar, 100 μm). (K) Bar graph showing extracellular lactate levels in day 20 DGUOK^+/+^ and DGUOK^Δ14/Δ5^ hepatocytes (mean ± SEM, n = 3, *p ≤ 0.05).

The phenotypes caused by DGUOK mutations in MTDPS3 patients have been well characterized. To determine whether the *DGUOK*^Δ14/Δ5^ hepatocyte-like cells faithfully mimic the hepatopathy in MTDPS3, we started by comparing the mtDNA levels across the time course of differentiation between wild-type and *DGUOK*^Δ14/Δ5^ cells using PCR (Bai and Wong, 2005; Dimmock et al., 2010). As expected, in control cells the mtDNA copy number increased more than 10-fold as the cells became fully differentiated at day 20 (Figure 3A). In contrast to the control cells, the levels of mtDNA found in the day 20 *DGUOK*^Δ14/Δ5^ hepatocytes were 5- to 10-fold less. In addition to PCR analysis, a recent study showed that staining with a low concentration of fluorescent DNA intercalating dyes could detect mtDNA in live cells (Sasaki et al., 2017). We therefore performed SYBR green I staining on the day 20 wild-type and *DGUOK*^Δ14/Δ5^ hepatocytes and qualitatively examined the cells by confocal microscopy. Although control iPSC-derived hepatocytes had bright punctate staining of the mitochondrial genomes, staining in DGUOK-deficient hepatocyte-like cells was markedly diminished (Figure S2A).

To ensure that the reduction of mtDNA was due specifically to the *DGUOK*^Δ14/Δ5^ allele and was not a consequence of off target effects of CRISPR/Cas9, we performed two independent rescue experiments. First, we generated *DGUOK*^Δ14/Δ5^ cells containing a wild-type *DGUOK* cDNA whose expression was doxycycline (Dox) dependent. These cells are referred to as *DGUOK*^Δ14/Δ5;indDGUOK^ (Figure S2B). Wild-type, *DGUOK*^Δ14/Δ5^, and DGUOK^Δ14/Δ5;indDGUOK^ cells were induced to differentiate to day 20 in the presence or absence of Dox (10 ng/mL), and the impact of expression of the *DGUOK* transgene on mtDNA levels was measured using PCR (Figure 3B). As before, mtDNA was dramatically reduced in *DGUOK*^Δ14/Δ5^ cells regardless of Dox. However, Although mtDNA levels were similarly reduced in DGUOK^Δ14/Δ5;indDGUOK^ hepatocyte-like cells cultured without Dox, the inclusion of Dox resulted in a complete recovery of mtDNA. Second, it has been reported that dAMP and dGMP supplementation can significantly increase mtDNA copy number in DGUOK-deficient fibroblasts (Taanman et al., 2003) and myotubes (Bulst et al., 2009). We therefore differentiated wild-type and *DGUOK*^Δ14/Δ5^ iPSCs in the presence and absence of 400μM dAMP and dGMP and measured mtDNA content by PCR at day 20. We found that including dAMP and dGMP during the culture of wild-type cells had no effect on mtDNA content. In contrast, treatment with dAMP and dGMP reversed mtDNA depletion in *DGUOK*^Δ14/Δ5^ cells (Figure 3C). From these data, we conclude that the loss of DGUOK activity causes mtDNA depletion in iPSC-derived hepatocyte-like cells.

Having demonstrated that *DGUOK* mutations recapitulate the reduction in mtDNA copy number seen in MTDPS3 patients, we next examined their impact on mitochondrial function. We examined mitochondrial structure in hepatocyte-like cells derived from either *DGUOK*^+/+^ or *DGUOK*^Δ14/Δ5^ iPSCs by electron microscopy (Figure 3D). Mitochondria in *DGUOK*^Δ14/Δ5^ hepatocytes showed decreased matrix density and abnormal cristae, which supports previous clinical reports (Dimmock et al., 2008b). Moreover, consistent with the disruption of mitochondrial structure observed by electron microscopy (EM), staining with tetramethylrhodamine ethyl ester (TMRE) showed a decrease in mitochondrial membrane potential in *DGUOK*^Δ14/Δ5^ hepatocytes compared with controls (Figure 3D). High-resolution images confirmed that the reduced TMRE signal was due to the loss of membrane potential, but not the reduction of mitochondria numbers (Figures S2C–S2E).

It has been reported that mtDNA depletion has to surpass a threshold to cause electron transport chain deficiencies (Durham et al., 2005). We therefore addressed whether the expression of electron transport chain genes was affected by the observed mtDNA depletion. We measured the levels of both nuclear-encoded and mitochondrial-encoded electron transport chain proteins in hepatocyte-like cells derived from control, *DGUOK*^Δ14/Δ5^, and DGUOK^Δ14/Δ5;indDGUOK^ iPSCs by immunoblot (Figure 3E). Whereas nuclear-encoded electron transport chain proteins were unaffected, the levels of mtDNA-encoded proteins from all electron transport chain complexes were reduced in *DGUOK*^Δ14/Δ5^ hepatocytes. Importantly, the same electron transport chain proteins were also depleted in *DGUOK*^Δ14/Δ5;indDGUOK^ hepatocytes in the absence of Dox, but they returned to levels found in the control cells after induction of the *DGUOK* transgene. Having confirmed the impact of DGUOK deficiency on the expression of mitochondrial electron transport chain genes, we next used a Seahorse bioanalyzer to study the function of mitochondria in control and DGUOK-deficient iPSC-derived hepatocyte-like cells. To exclude the possibility that DGUOK deficiency may have an impact on total cellular protein levels, which is used for normalization of the Seahorse assay, we confirmed that the average protein content in wild-type and *DGUOK*^Δ14/Δ5^ hepatocyte-like cells was the same (Figure S2F). When OCR was measured, it revealed a significant reduction in oxidative phosphorylation activity in *DGUOK*^Δ14/Δ5^ hepatocytes compared with controls (Figure 3F). Hepatocytes have high microsomal and peroxisome activities as well as multiple P450-dependent reactions, all of which contribute to oxygen consumption that is independent of the mitochondria (Harper et al., 2008). To obtain an accurate assessment of mitochondrial respiration rates, the data were therefore corrected to the baseline level (non-mitochondrial) OCR. Both basal and maximum OCR were dramatically reduced by the loss of DGUOK function (Figures 3G and 3H), indicating a lower level of basal oxidative phosphorylation and an attenuated capacity for energy production.

Because the reduction in mitochondrial respiration in the *DGUOK*^Δ14/Δ5^ hepatocytes implied that these cells would be deficient in ATP production, we measured total ATP levels using a luciferase assay in wild-type, *DGUOK*^Δ14/Δ5^, and *DGUOK*^Δ14/Δ5;indDGUOK^ iPSC-derived hepatocytes in the absence or presence of Dox (Figure 3I). As predicted, the loss of DGUOK resulted in a 40% reduction in intracellular ATP levels in DGUOK-deficient hepatocytes, and this impact could be fully rescued by inducing expression of the *DGUOK* transgene in the *DGUOK*^Δ14/Δ5;indDGUOK^ hepatocytes. Moreover, when mitochondrial ATP production was measured after culture of cells in galactose as a primary carbon source, the impact of loss of DGUOK on cellular ATP levels was exacerbated (Figure S2G). Mitochondrial function is essential for redox regulation, so we also quantified the impact of DGUOK deficiency on the levels of reactive oxygen species (ROS) using a redox-sensitive fluorescent probe (DCFDA) (Figure 3J). Although in the context of the mtDNA depletion, oxidative phosphorylation activity is reduced, our results revealed an increase in ROS levels in the DGUOK-deficient hepatocyte-like cells, as has been described for other types of mtDNA depletion syndromes (Antonenkov et al., 2015). Although ROS levels increased in the absence of DGUOK, we found no significant difference in expression the ROS scavenger protein SOD2 between wild-type and *DGUOK*^Δ14/Δ5^-derived hepatocytes (Figure S2H). We also determined levels of the mitophagy marker PINK1 and found it to be unaffected by loss of DGUOK (Figure S2H). It is known that most MTDPS3 patients have lactic acidosis that is caused by the increased rates of glycolysis (Almannai et al., 2018). We therefore compared the extracellular lactate levels in control and *DGUOK*^Δ14/Δ5^ hepatocytes and found that lactate levels were significantly increased in the DGUOK-deficient hepatocytes compared with controls (Figure 3K). Cumulatively, our results demonstrate that hepatocytes generated from *DGUOK*^Δ14/Δ5^ iPSCs faithfully recapitulate the pathophysiology of MTDPS3.

### A Drug Screen Reveals that NAD Treatment Rescues Mitochondrial Dysfunction and ATP Levels in DGUOK^Δ14/Δ5^ Hepatocyte-like Cells

**Figure 4.**
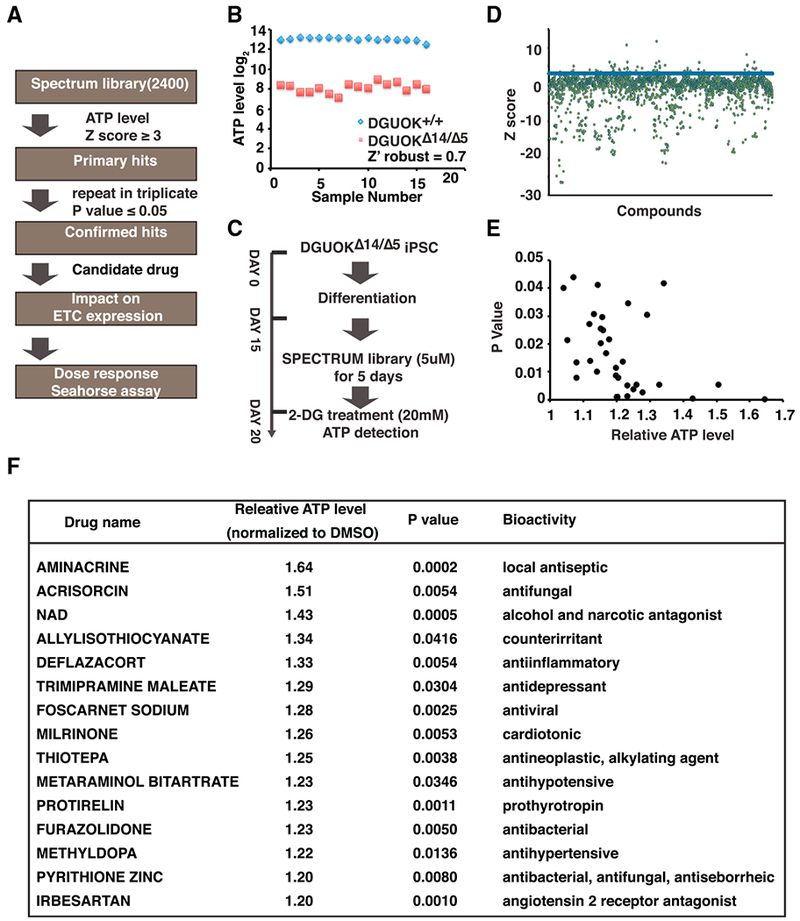
Drug Screen Using DGUOK-Deficient iPSC-Derived Hepatocyte-like Cells (A) Flow chart showing approach to identify drugs. (B) Graphs showing the ATP levels in DGUOK^+/+^ (blue diamond) and DGUOK^Δ14/Δ5^ (red box) cells on day 20 of differentiation. Results were used to calculate Z factor (Z′_robust_ = 0.7). (C) Schematic showing the experimental approach used in the primary screen. (D) Graphs showing the result of primary screen. *Z* scores were calculated on the basis of ATP levels. Drugs with *Z* scores ≥ 3 (blue bar) were identified as primary hits. (E) Graph showing relative levels of ATP (normalized to control wells) of confirmed hits (p ≥ 0.05). (F) Table showing a list of top 15 confirmed hits with increases in ATP levels ≥ 20%.

Currently, no curative treatment is available for MTDPS3, and efforts to develop treatments are limited because of the small patient pool. Previously, our lab has successfully established a platform using iPSC-derived hepatocytes that supports small molecule screens (Cayo et al., 2017; Jing et al., 2017). We, therefore, reasoned that a similar approach could be applied to identify potential therapeutics for MTDPS3 (Figure 4A). Because the symptoms of MTDPS3 are ultimately a consequence of the cell’s loss of mitochondrial function, we predicted that drugs that restore ATP levels in *DGUOK*^Δ14/Δ5^ hepatocytes could offer a potential treatment. Therefore, we used a luciferase ATP assay to identify primary hits. The suitability of an assay for high-throughput screening is most commonly determined by establishing the Z factor (Zhang et al., 1999). A reading of 1.0 is a perfect assay, and ≥0.5 is considered excellent. We tested the feasibility of using the luciferase ATP assay for high-throughput screening by determining its ability to detect differences in ATP content between control and *DGUOK*^Δ14/Δ5^ hepatocytes and calculating the Z factor (Figure 4B). The results yielded a Z′_robust_ of 0.7, indicating that the assay is highly compatible with high-throughput screening. We next performed a primary screen to identify hits that increased cellular ATP levels. *DGUOK*^Δ14/Δ5^ iPSCs were plated in 30 96-well plates, differentiated into hepatocyte-like cells, and treated with approximately 2,400 drugs (5 μM) from the SPECTRUM collection library from day 15 to day 20 of the differentiation (Figure 4C). We chose the SPECTRUM collection for our screening because the majority of drugs (~1,300) in this library have been approved for human use in the United States, Europe, or Japan. Any identified drugs could therefore be repurposed and rapidly advance to clinical trials. Drugs that increase glycolytic ATP production will likely increase acidosis-associated etiology and are consequently not suitable for clinical application. To circumvent this potential problem and to focus on drugs that improved oxidative phosphorylation, glycolysis was inhibited by culturing the cells in the presence of 2-deoxy-D-glucose (2-DG) for 24 hr, prior to performing the assay. ATP levels were then used to identify compounds that increase cellular energy production.

To analyze the results of the primary screen, data from each well was collected and converted to a *Z* score on the basis of distribution per plate (Table S1). Drugs that resulted in *Z* scores ≥ 3 were considered for further analyses (Figure 4D). All the primary hits were repeated in triplicate for statistical analysis, and 34 drugs were confirmed to reproducibly (p ≤ 0.05) increase ATP levels in *DGUOK*^Δ14/Δ5^ hepatocyte-like cells (Figure 4E; Table S1). Among the 34 hits, 15 increased ATP levels compared with control wells by ≥20% (Figure 4F). We performed a bioinformatics analysis using STITCH to identify proteins targeted by each of the 34 drugs (Szklarczyk et al., 2016). Gene Ontology (GO) analysis of the biological processes associated with the target proteins showed that a plethora of metabolic processes known to control cellular energy production, such as the tricarboxylic acid (TCA) cycle, fatty acid oxidation (FAO), and electron transport chain, were highly overrepresented (Figure S3). We next asked whether any of the drugs could increase the expression of mtDNA-encoded electron transport chain subunits. qRT-PCR was performed using mRNA samples from *DGUOK*^Δ14/Δ5^ hepatocyte-like cells treated with vehicle (DMSO) or drugs (5 μM) to determine the expression of representative mtDNA-encoded electron transport chain subunits from complexes I, III, IV, and V (ND1, cytochrome b, MTCO1, and ATP8). We found that several of the drugs were capable of increasing the expression of one or more of the electron transport chain subunits (Figure S4).

**Figure 5.**
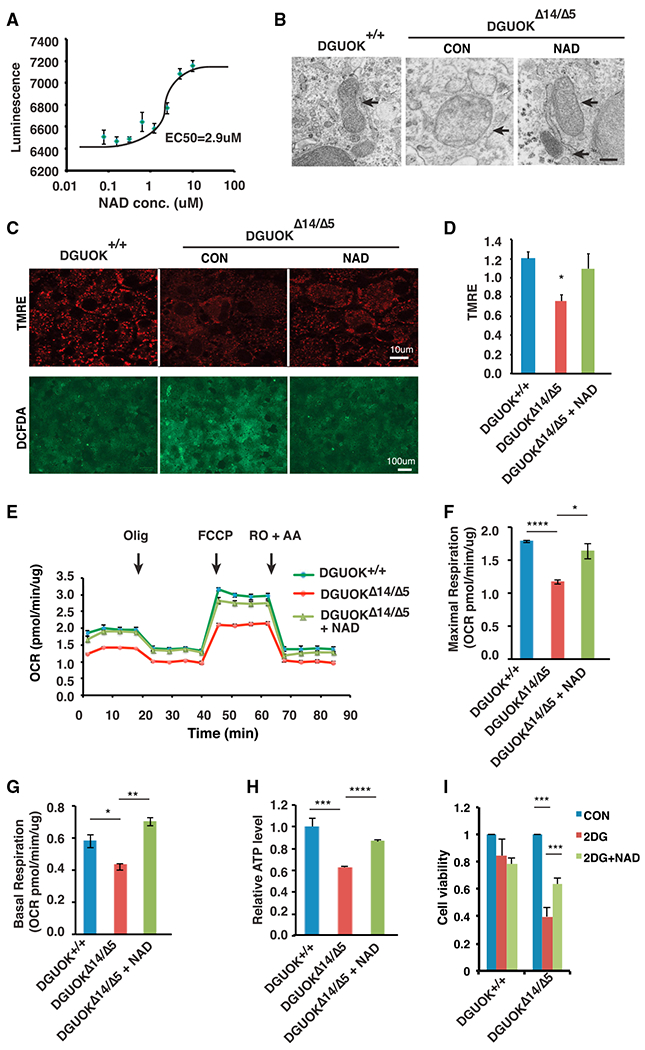
NAD Is Capable of Restoring Mitochondrial Activity and ATP Levels (A) Graph showing the result of a dose-response assay to detect ATP levels (luminescence) in DGUOK^Δ14/Δ5^ hepatocyte-like cells treated with NAD at 0.08, 0.16, 0.32, 0.6, 1.25, 2.5, 5, and 10 μM (n = 4 biological replicates, mean ± SEM). (B) Representative electron microscopy images of mitochondria in DGUOK^+/+^, untreated DGUOK^Δ14/Δ5^, and NAD (5 μM) treated DGUOK^Δ14/Δ5^ hepatocyte-like cells. Arrow, mitochondria. Scale bar, 200 nM. (C) Micrographs showing TMRE (red) and DCFDA (green) staining of day 20 DGUOK^+/+^ and DGUOK^Δ14/Δ5^ hepatocytes. Scale bars, 100 μm (lower) and 10 μm (upper). (D) Bar graph showing relative TMRE staining intensity measured by plate reader (n = 8, mean ± SEM, *p ≤ 0.05). (E) Graph showing OCR in wild-type, untreated DGUOK^Δ14/Δ5^, and NAD (5 μM) treated DGUOK^Δ14/Δ5^ hepatocyte-like cells, determined by seahorse assay. (F and G) Maximal respiration (F) and basal respiration (G) were calculated on the basis of OCR (n = 3 biological replicates, mean ± SEM, *p ≤ 0.05, **p ≤ 0.01, ****p ≤ 0.0001). (H) Bar graph showing relative ATP levels in wild-type cells, untreated DGUOK^Δ14/Δ5^ hepatocyte-like cells, and NAD (5 μM) treated DGUOK^Δ14/Δ5^ hepatocyte-like cells (n = 8 biological replicates, mean ± SEM, ****p ≤ 0.0001). (I) Bar graph showing cell viability of DGUOK^+/+^ and DGUOK^Δ14/Δ5^ hepatocyte-like cells that were untreated (blue) or treated for 24 hr with 20 mM 2-DG (red) or 2-DG + 5 μM NAD (green) (n = 3, mean ± SEM, *p % 0.05, ***p ≤ 0.001).

Of the drugs examined, we found that NAD could reproducibly increase ATP levels and increase the expression of all mitochondrial-encoded electron transport chain genes examined (Figure S4). Moreover, as a bioactive form of niacin, NAD has minimal toxicity, which could be desirable for long-term administration to patients. To determine the efficacy of NAD, we first performed an eight-point dose-response assay with concentrations in NAD ranging from 0.08 to 10 μM. Increased doses of NAD led to a classic sigmoidal increase of ATP levels in DGUOK-deficient iPSC-derived hepatocyte-like cells with a half maximal effective concentration (EC_50_) of 2.9 μM (Figure 5A). Because we had shown that NAD was able to enhance the expression of electron transport chain genes, we asked whether NAD treatment rescues mitochondrial dysfunction. We examined mitochondria structure using electron microscopy and found that in contrast to the untreated *DGUOK*^Δ14/Δ5^ hepatocyte-like cells, NAD-treated *DGUOK*^Δ14/Δ5^ cells exhibited normal morphology with increased mitochondria matrix density and normal cristae structure (Figure 5B). To test whether the restored mitochondrial structure leads to improved mitochondria function, we performed TMRE staining followed by confocal microscopy, which qualitatively revealed an increase of mitochondrial membrane potential in *DGUOK*^Δ14/Δ5^ hepatocytes after treatment with NAD (5 μM) (Figure 5C). A quantitative measurement of TMRE staining in multiple samples confirmed that the levels of mitochondrial membrane potential are comparable between wild-type cells and NAD-treated DGUOK-deficient cells (Figure 5D). NAD treatment could also efficiently reduce the ROS levels, detected by DCFDA staining, in the DGUOK-deficient hepatocytes (Figure 5C). Furthermore, by measuring OCR in wild-type and *DGUOK*^Δ14/Δ5^ hepatocyte-like cells cultured with or without NAD treatment, we revealed that NAD improved the oxidative phosphorylation activity of *DGUOK*^Δ14/Δ5^ hepatocytes (Figure 5E), achieving both maximal respiration (Figure 5F) and basal respiration capacities (Figure 5G) close to those observed in wild-type cells. Finally, we addressed whether the observed beneficial effects of NAD treatment on mitochondria activity and electron transport chain gene expression are sufficient to rescue the DGUOK mutation-associated ATP deficits. By comparing the ATP levels in wild-type and *DGUOK*^Δ14/Δ5^ cells treated with vehicle or NAD, we found that after NAD treatment the *DGUOK*^Δ14/Δ5^ hepatocyte-like cells no longer showed a significant reduction of ATP levels compared with wild-type cells (Figure 5H).

To evaluate the kinetics of NAD action, we treated DGUOK-deficient cells with 5μM NAD for increasing lengths of time and collected cells at day 20 of differentiation to determine ATP levels (Figure S5A). NAD increased ATP as early as 24 hr of treatment until a maximum impact was reached at 3 days of treatment. We also examined the impact of NAD on ATP levels after the completion of differentiation. When DGUOK-deficient cells were treated with NAD between days 20 through 25, ATP production again significantly increased (Figure S5B).

MTDPS3 patients suffer from progressive liver failure, implying that that loss of DGUOK should affect hepatocyte viability. However, under standard culture conditions, which contain glucose (25 mM), loss of DGUOK did not affect the number of hepatocyte-like cells. We reasoned that any requirement for oxidative phosphorylation could be circumvented if ATP was produced by the conversion of glucose to pyruvate through glycolysis. We therefore examined the impact of loss of DGUOK on cell viability when glycolysis was inhibited by 2-DG (Figure 5I). After 48 hr of treatment with 2-DG, *DGUOK*^+,+^ iPSC-derived hepatocytes remained viable, and there was no significant change in cell number compared with untreated cells. In contrast, when *DGUOK*^Δ14/Δ5^ hepatocytes were cultured with 2-DG, cell viability was compromised. Finally, when treated with NAD, there was a significant improvement in viability *DGUOK*^Δ14/Δ5^ hepatocytes cultured in 2-DG (Figure 5I). Cumulatively, these data are consistent with the view that treatment of DGUOK-deficient hepatocytes with NAD improves viability by improving mitochondrial function.

### NAD Rescues DGUOK Deficiency through the Activation of PGC1α

**Figure 6.**
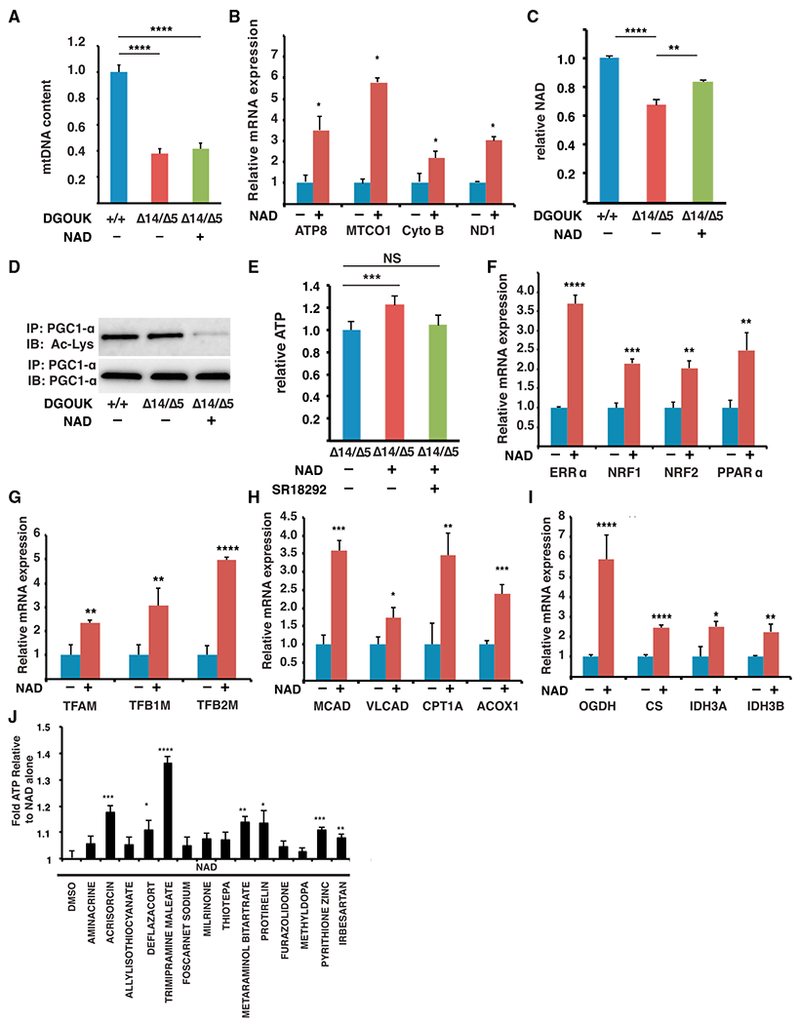
NAD Restores ATP Levels through Activation of PGC1α (A) Bar graph showing relative mtDNA content in DGUOK^+/+^ cells, untreated DGUOK^Δ14/Δ5^ hepatocyte-like cells, and NAD-treated DGUOK^Δ14/Δ5^ hepatocyte-like cells (n = 8 biological replicates, mean ± SEM, ****p ≤ 0.0001) (B) Bar graph showing levels of mRNAs encoding electron transport chain proteins in DGUOK^Δ14/Δ5^ hepatocyte-like cells in the presence and absence of NAD (5 μM) (mean ± SEM, *p ≤ 0.05). (C) Bar graph showing that NAD treatment increases intracellular NAD^+^ levels in DGUOK^Δ14/Δ5^ hepatocyte-like cells (n = 8 biological replicates, mean ± SEM, ****p ≤ 0.0001). (D) Micrograph showing the results of an immunoprecipitation (anti-PGC1α) followed by immunoblot to detect total PGC1α and acetylated PGC1α in DGUOK^+/+^ iPSC-derived hepatocytes and in DGUOK^Δ14/Δ5^ hepatocyte-like cells with and without 5 μM NAD treatment. (E) Bar graph showing relative ATP levels in control DGUOK^+/+^ hepatocyte-like cells and in DGUOK^Δ14/Δ5^ hepatocyte-like cells treated with NAD in the absence and presence of SR18292 (n = 8, mean ± SEM, ***p ≤ 0.001). (F) Bar graph showing relative steady-state mRNA levels encoding transcription factors regulated by PGC1α (ERRα, NRF1, NRF2, and PPARα) (n = 3 biological replicates, mean ± SD, **p ≤ 0.01, ***p ≤ 0.001, ****p ≤ 0.0001). (G) Bar graph showing relative steady-state mRNA levels encoding mitochondrial transcription factors (TFAM, TFB1M, and TFB2M) (n = 3 biological replicates, mean ± SD, **p ≤ 0.01, ****p ≤ 0.0001). (H) Bar graph showing relative steady-state mRNA levels encoding FAO enzymes (MCAD, VLCAD, CPT1A, and ACOX1) (n = 3 biological replicates, mean ± SD, *p ≤ 0.05, **p ≤ 0.01, ***p ≤ 0.001). (I) Bar graph showing relative steady-state mRNA levels encoding key TCA cycle enzymes (OGDH, CS, IDH3A, and IDH3B) (n = 3 biological replicates, mean ± SD, *p ≤ 0.05, **p ≤ 0.01, ****p ≤ 0.0001). (J) Bar graph showing relative ATP levels in DGUOK^Δ14/Δ5^ hepatocyte-like cells treated with 5 μM NAD alone (DMSO) or with 5 μM NAD + 5 μM of each drug identified in the primary screen to increase ATP ≥ 20% (n = 8 biological replicates, mean ± SD, *p ≤ 0.05, **p ≤ 0.01, ***p ≤ 0.001, ****p ≤ 0.0001).

To determine the mechanism through which NAD rescues DGUOK-associated mitochondrial dysfunction, we first asked if NAD restores mtDNA content in DGUOK-deficient hepatocytes. Surprisingly, the mtDNA copy number in NAD-treated *DGUOK*^Δ14/Δ5^ hepatocytes was comparable with that in untreated cells (Figure 6A), suggesting that NAD regulates mitochondrial functions through mechanisms other than restoring mtDNA levels. As discussed earlier, treatment of *DGUOK*^Δ14/Δ5^ iPSC-derived hepatocytes with NAD resulted in increased levels of mtDNA-encoded mRNAs, including those encoding electron transport chain proteins (Figure 6B). The observed elevation in mitochondrial mRNA levels suggests that NAD therefore influences mitochondrial gene expression. It has been reported that NAD promotes mitochondrial function by activating PGC1α through deacetylation by Sirtuin 1 (Sirt1) (Nemoto et al., 2005; Mouchiroud et al., 2013). PGC1α functions as a key regulator of cellular metabolism by co-activating a variety of transcription factors that are essential for mitochondria function and energy production, including estrogen-related receptor alpha (ERRα), peroxisome proliferator-activated receptor alpha (PPARα), and nuclear respiratory factors (NRF1 and NRF2) (Wu et al., 1999; Vega et al., 2000; Schreiber et al., 2003). We therefore speculated that NAD-mediated elevation of ATP levels in DGUOK-deficient hepatocytes might be attributed to the activation of PGC1α pathways. To test this, we first measured the intracellular NAD^+^ levels in wild-type, untreated, and NAD-treated *DGUOK*^Δ14/Δ5^ hepatocyte-like cells. Figure 6C shows that NAD levels were lower in DGUOK-deficient cells compared with control cells, and treatment of NAD from day 15 to day 20 significantly increased intracellular NAD^+^ in *DGUOK*^Δ14/Δ5^ cells. We next determined whether the increase in NAD^+^ leads to PGC1α activation through deacetylation. We performed PGC1α immunoprecipitation in wild-type cells, untreated *DGUOK*^Δ14/Δ5^ hepatocytes, and NAD-treated *DGUOK*^Δ14/Δ5^ hepatocytes and immunoblotted with an antibody that detects acetylated lysine. We found that although the total protein levels of PGC1α were unaffected by NAD treatment, the portion of PGC1α that was acetylated was greatly reduced in NAD-treated *DGUOK*^Δ14/Δ5^ hepatocytes, resulting in a substantial increase in the relative level of active PGC1α (Figure 6D). To confirm that the effect of NAD is mediated by the activation of PGC1α, we treated the *DGUOK*^Δ14/Δ5^ hepatocytes with NAD in the presence of a PGC1α inhibitor SR18292 (Sharabi et al., 2017). Figure 6E shows that SR18292 could block NAD from increasing the ATP levels in DGUOK-deficient hepatocytes. Next, we asked whether the increase in PGC1α activity leads to enhanced expression of genes implicated in energy metabolism. Studies have shown that PGC1α not only binds to ERRα, PPARα, and NRFs as a coactivator but also forms autoregulatory loops with these partner transcription factors to regulate their expression (Wu et al., 1999; Schreiber et al., 2003; Huss et al., 2004). We therefore determined the mRNA levels encoding ERRα, PPARα, and NRFs by qRT-PCR and revealed that NAD treatment increased the mRNAs encoding all four of these transcription factors in *DGUOK*^Δ14/Δ5^ hepatocytes (Figure 6F). We also examined the downstream effects of the activation of these transcription factors. ERRα and NRF are known to upregulate mitochondria biogenesis by increasing the levels of mitochondrial transcription factors that are responsible for transcription of the mtDNA-encoded genes (Wu et al., 1999; Gleyzer et al., 2005). We therefore determined the levels of mRNAs encoding the three major mitochondrial transcription factors: mitochondria transcription factor A (TFAM), mitochondria transcription factor B1 (TFB1M), and mitochondria transcription factor B2 (TFB2M) in *DGUOK*^A14/A5^ hepatocytes treated with vehicle or NAD. As shown in Figure 6G, treatment with NAD increased their mRNA levels between 2- and 5-fold, indicating enhanced transcription of the mitochondrial genome. Because ERRα and PPARα play essential roles in regulating glucose and fatty acid metabolism, we measured the levels of mRNAs encoding key enzymes of FAO (MCAD, VLCAD, CPT1A, and ACOX1) and the TCA cycle (OGDH, CS, IDH3A, and IDH3B) (Figures 6H and 6I). Our results revealed a significant increase in expression of genes encoding of all these enzymes, indicating an increase in fatty acid/glucose metabolism, which also contributes to the increased ATP levels by fueling the mitochondria. Interestingly, no increase in ATP levels or activation of PGC1α pathways was observed in wild-type iPSC-derived hepatocytes treated with NAD (Figures S5C and S5D).

Finally, we noted that most of the other drugs identified in the primary screen had no effect on the level of mRNAs encoding electron transport chain proteins (Figure S4), which implies that they act through a mechanism that is different from that of NAD. With this in mind, we examined whether any of the drugs acted synergistically with NAD to increase ATP levels. DGUOK-deficient hepatocytes were treated with each of the drugs in the absence or presence of NAD and the impact on ATP levels were measured as before. Of the 14 drugs that were examined, 7 significantly boosted ATP production in DGUOK-deficient hepatocytes over NAD treatment alone (Figure 6J). We conclude that NAD rescues DGUOK-associated mitochondrial dysfunction and ATP depletion by PGC1α-mediated enhancement of mitochondrial gene expression and energy metabolism. Moreover, the level that NAD can increase ATP production can be enhanced by combining its action with other drugs identified in the screen.

### Nicotinamide Riboside Increases ATP Production in DGUOK-Deficient Rats

**Figure 7.**
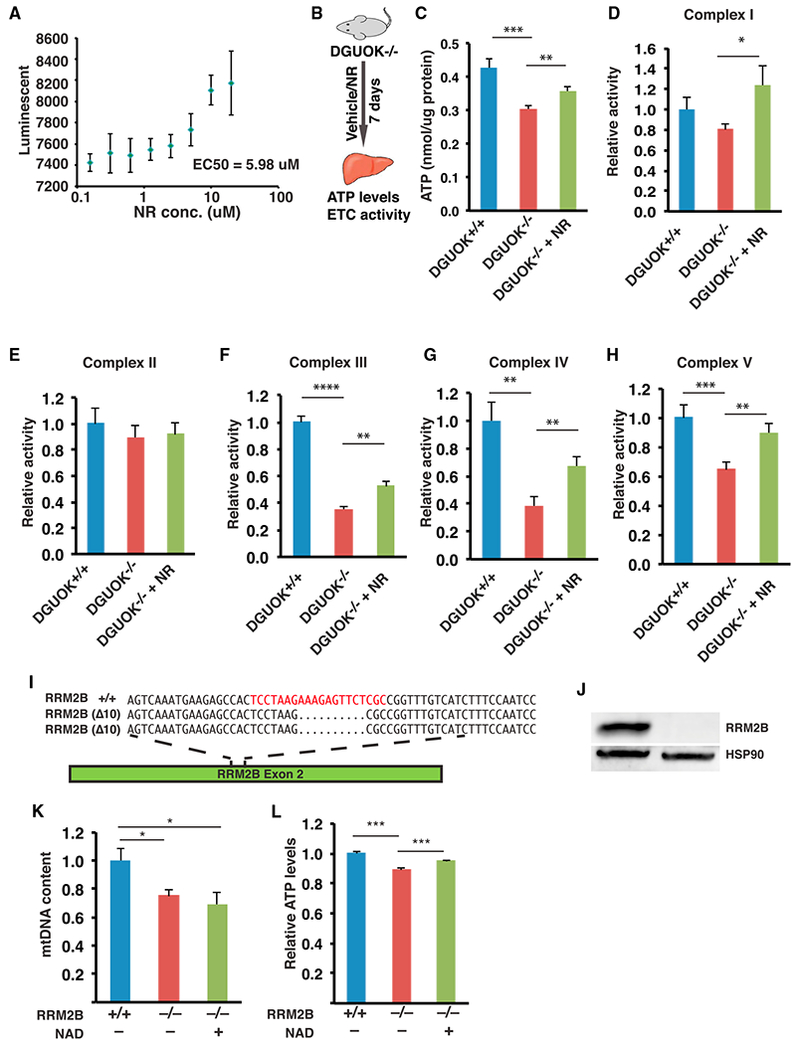
NR Increases Mitochondrial Activity and ATP Levels in DGUOK-Deficient Rats and RRM2B-Deficient Hepatocyte-like Cells (A) Graph showing the result of a dose-response assay to detect ATP levels (luminescence) in DGUOK^Δ14/Δ5^ hepatocyte-like cells treated with nicotinamide riboside (NR) at 0.08, 0.16, 0.32, 0.6, 1.25, 2.5, 5, and 10 μM (n = 4 biological replicates, mean ± SEM). (B) Scheme of experimental approach used to test the effect of NR in DGUOK-deficient rats. (C–H) Bar graphs showing relative ATP levels (C), and electron transport chain complex I (D), II (E), III (F), IV (G), and V (H) activities in livers from DGUOK^+/+^ (n = 6, blue) or DGUOK^−/−^ rats without (n = 6, red) or with 500 mg/kg/day NR treatment for 7 days (n = 7, green) (mean ± SEM, **p ≤ 0.01, ***p ≤ 0.001) (I) Schematic illustration of *RRM2B* exon 2 showing the CRISPR/Cas9 guide (red) and nucleotide sequences of *RRM2B* wild-type (RRM2B^+/+^) and mutant alleles (RRM2B^Δ10/Δ10^). (J) Micrograph of an immunoblot to detect expression of RRM2B and HSP90 in RRM2B^+/+^ and RRM2B^Δ10/Δ10^ iPSC–derived hepatocytes. (K) Bar graph showing relative mtDNA content in wild-type cells, untreated RRM2B^−/−^ hepatocyte-like cells, and NAD-treated RRM2B^−/−^ hepatocyte-like cells (n = 6, mean ± SEM, *p ≤ 0.05). (L) Bar graph showing relative ATP levels in control RRM2B^+/+^ iPSC-derived hepatocytes and in RRM2B^−/−^ hepatocyte-like cells untreated or treated with 5 mM NAD (n = 12, mean ± SEM, ***p ≤ 0.001).

To evaluate the potential of NAD as a treatment for MTDPS3, we asked whether NAD could also rescue the mitochondria dysfunction and ATP reduction associated with the mutation of DGUOK *in vivo*. A DGUOK-deficient rat model has been previously reported (Bennett et al., 2016). Although the rats are viable and do not develop progressive liver failure, they exhibit a significant reduction of hepatic mitochondrial activity and ATP levels. We therefore decided to test the effect of NAD in DGUOK-deficient rats. Although it is known that cultured mammalian cells are capable of directly taking up NAD through carriers that are yet to be identified, studies have shown that orally administrated NAD is rapidly hydrolyzed in the small intestine (Billington et al., 2008). As a result, an alternative regimen is required to boost intracellular NAD levels *in vivo*. Multiple precursors or intermediates of NAD metabolism, such as nicotinic acid (NA), nicotinamide (NAM), and nicotinamide riboside (NR), are known to be capable of elevating the production of NAD. Among them, NR has been reported to possess superior pharmacokinetics and bioavailability (Trammell et al., 2016). Studies have shown that NR-treated animals have improved mitochondria function due to the NAD^+^-mediated SIRT1 activation (Canto et al., 2012; Cerutti et al., 2014). Therefore, we decided to use NR to increase intracellular NAD^+^ levels in *DGUOK*^−/−^ rats. We first validated the efficiency of NR in elevating NAD in *DGUOK*^Δ14/Δ5^ iPSC-hepatocytes. A dose response assay performed on *DGUOK*^Δ14/Δ5^ hepatocyte-like cells suggested that, similar to NAD, NR treatment also led to increased ATP levels (Figure 7A). We next examined whether NR could increase mitochondrial function and ATP levels in *DGUOK*^−/−^ rats. Thirteen DGUOK-mutant rats (8 weeks old) were orally provided vehicle (n = 6) or NR (500 mg/kg/day, n = 7) for 1 week. Livers were collected and used to analyze ATP levels and mitochondrial function (Figure 7B). The result of ATP assays revealed that ATP levels in the livers of *DGUOK*^−/−^ rats were reduced compared with controls and could be significantly improved after 1 week of NR treatment (Figure 7C). To test whether this increase was caused by the improvement of mitochondrial function, we next determined the activities of each complex in the electron transport chain. As expected, loss of DGUOK impaired electron transport chain complex activity; however, when treated with NR, the activities of electron transport chain complexes I, III, IV, and V, all of which contain mtDNA-encoded subunits, significantly improved (Figures 7D and 7F–7H). In contrast, the activity of complex II, which is solely encoded by nuclear genes and unaffected by DGUOK mutations (Bennett et al., 2016), was not significantly enhanced (Figure 7E). We conclude that NR treatment of DGUOK-deficient rats increases mitochondria function and improves hepatic ATP levels *in vivo*.

### NAD Treatment Improves ATP Levels in RRM2B-Deficient Hepatocyte-like Cells

Given the observation that NAD rescues ATP production in *DGUOK*^Δ14/Δ5^ iPSC hepatocytes, we asked whether NAD treatment could benefit a broader range of mtDNA depletion syndromes. Recently, mtDNA depletion syndromes received international attention (Huxtable, 2018) because of the death of an infant who had mutations in the *RRM2B* gene encoding the ribonucleotide reductase regulatory TP53 inducible subunit M2B. RRM2B is part of the ribonucleotide reductase complex that catalyzes the conversion of ribonucleoside diphosphates into deoxyribonucleoside diphosphates (Cho and Yen, 2016). Disruption of RRM2B causes MTDPS8A/B, which results in a range of clinical features including neurological impairment, muscle weakness, and liver damage (Valencia et al., 2016; Bourdon et al., 2007). We therefore generated *RRM2B*^−/−^ iPSCs using CRISPR/Cas9 (Figures 7I and 7J). Examination of the mtDNA content and ATP levels after differentiation to hepatocyte-like cells revealed that both were significantly diminished, although less severely than was observed for *DGUOK*^Δ14/Δ5^ iPSC hepatocytes (Figures 7K and 7L). Despite the milder phenotype, treatment of *RRM2B*^−/−^ hepatocyte-like cells with NAD significantly improved ATP production without affecting mtDNA levels (Figures 7K and 7L). These data imply that the beneficial effects of NAD are not restricted to DGUOK deficiency and could be used to treat a broad range of mtDNA depletion syndromes.

## DISCUSSION

More than three decades have passed since the earliest cases of mtDNA depletion syndrome were reported, and hundreds of patients with a variety of mtDNA depletion-causing mutations have subsequently been identified. Such syndromes have recently received international attention after an infant named Charlie Gard became the subject of a ‘‘best interests’’ case in the United Kingdom in which the parents and health care providers disagreed over termination of life support (Huxtable, 2018). Unfortunately, no curative therapy is available for any of these disorders, and the discovery of treatments is impeded by the difficulties in obtaining patient samples and the absence of animal models that fully mimic the pathology of mtDNA depletion syndrome. Recently, human pluripotent stem cells combined with gene-editing techniques have paved the way to identify treatments for rare diseases in culture, without the need to access patients directly (Robinton and Daley, 2012; Swinney and Xia, 2014; Ding et al., 2013; Cayo et al., 2012). In this study, we successfully used CRISPR/Cas9 gene editing to generate iPSC-derived hepatocytes that model the most prevalent hepatic type of mtDNA depletion syndrome, MTDPS3.

Previous efforts in identifying mtDNA depletion syndrome treatments have focused on reversing the pathogenesis by trying to rescue mtDNA depletion (Bulst et al., 2009; Cámara et al., 2014). However, the small number of biological processes that are involved in the regulation of mtDNA homeostasis makes it difficult to rationally find ‘‘druggable’’ targets. In the present study, we used a phenotypic screen to identify drugs that are capable of increasing ATP levels in DGUOK-deficient hepatocytes. As the clinical manifestations of MTDPS3 are primarily caused by unmet energy requirements, drugs that rescue the DGUOK-associated reduction of ATP will hold promise. Most important, such a strategy allows us to screen all the signaling pathways or biological processes that affect cellular ATP levels and thus greatly broadens the spectrum of the target pool. As a result, we were able to successfully identify NAD as a lead drug that could promote mitochondrial function and restore ATP without reverting the reduction of mtDNA copy number in DGUOK-deficient hepatocytes. Our study showed that NAD treatment caused an elevation of intracellular NAD^+^, which promoted SIRT1-mediated activation of PGC1α. PGC1α is a master regulator of energy production that indirectly increases the transcription of mtDNA-encoded electron transport chain subunits and enhances fatty acid and glucose metabolism, which further fuels the electron transport chain for mitochondrial ATP production. Our finding that NAD also increased ATP production by iPSC-derived hepatocytes with mutations in *RRM2B* implies that the potential of NAD as a treatment may not be limited to DGUOK-related MTDPS3.

NAD homeostasis plays an important role in cellular energy metabolism, and repletion of intracellular ATP by treatment with NAD or NAD precursors has showed beneficial effects on a wide range of metabolic and age-related disorders (Canto et al., 2012; Gomes et al., 2013; Scheibye-Knudsen et al., 2014). However, the ability of NAD to rescue mitochondrial function in mtDNA depletion disorders has not previously been appreciated and emphasizes the benefits of using a phenotypic screen. Whether, NAD will be effective in patients will require clinical trials; however, our data demonstrate that enhancing NAD^+^ levels in a DGUOK-deficient rat model using a NAD precursor, NR, increases hepatic mitochondria activity and ATP levels. Although NR has been widely used *in vivo* to increase NAD^+^ levels because of its oral bioavailability (Cerutti et al., 2014; Zhang et al., 2016; Trammell et al., 2016), other NAD precursors, such as nicotinamide and NMN, are also known to be capable of elevating NAD^+^ levels and hold promise for the treatment of metabolic and neurodegenerative diseases (Yoshino et al., 2011; Yang et al., 2014; Long et al., 2015). NAD has minimal toxicity and thus can be administrated long term at relatively high dose without causing severe adverse events (Knip et al., 2000; Conze et al., 2016). In our experiments, the DGUOK-deficient cells and animals were treated with NR for a relatively short period (5 days in cells and 7 days in rats). We believe it likely that chronic long-term exposures that use a combination of NAD along with other drugs identified in our screen could have an even greater beneficial effect.

Although liver disease is one of the primary clinical features and cause of death of MTDPS3, some of the DGUOK mutations lead to multisystem disorders with muscular or neurological manifestations, including hypotonia, psychomotor retardation, or nystagmus (Dimmock et al., 2008a, 2008b). It is desirable to test the impact of NAD repletion on the mitochondrial function and ATP levels in brain or muscle. Unfortunately, the DGUOK-deficient rats showed no biochemical or phenotypical abnormalities in tissues other than liver, preventing us from studying the therapeutic potential of NAD for multi-organ DGUOK disorders in this model. Nevertheless, it has been reported that NR treatment led to an increase of NAD^+^ in skeletal muscle and brown adipose tissue and showed a protective role against mitochondrial myopathy in mice (Khan et al., 2014). Additionally, nicotinamide has been shown to be able to cross the blood-brain barrier and increase NAD levels in the brain (Spector and Johanson, 2007); the effects of NR and NMN on improving mitochondrial function in neural system are supported by multiple studies using animal models (Gong et al., 2013; Long et al., 2015). On the basis of these studies, it is possible that NAD is capable of rescuing the muscular or neurological manifestations of MTDPS3 and holds promise in the treatment of other forms of mtDNA depletion syndromes that primarily affect brain and muscle. It will be of interest to understand the effects of NAD treatment on various cell types, such as myocytes or neurons, differentiated from iPSCs with MTDPS-associated mutations.

It is important to note that other drugs identified by our screen that could lead to treatment of mtDNA depletion syndromes in addition to NAD. For instance, trimipramine was found to synergize with NAD to further enhance ATP production by DGUOK-deficient cells (Figure 6J). Trimipramine is a tricyclic antidepressant used to treat depression (Berger and Gastpar, 1996). Another confirmed hit, deflazacort, is a glucocorticoid used as an anti-inflammatory drug. Glucocorticoids are known to stimulate mitochondria biogenesis (Weber et al., 2002), and deflazacort was recently approved by the U.S. Food and Drug Administration to treat Duchenne muscular dystrophy, which in addition to the inflammatory disease is known to be associated with mitochondrial dysfunction (Vila et al., 2017). Taken together, the phenotypic screen using iPSC-derived DGUOK-deficient hepatocytes provides a variety of new perspectives in the identification of therapies for mtDNA depletion syndromes. We believe that a similar strategy can be applied for drug discovery or for mechanistic dissection of other rare diseases that have previously been difficult to study.

## STAR★METHODS

### KEY RESOURCES TABLE

**Table:** 

REAGENT or RESOURCE	SOURCE	IDENTIFIER
Antibodies		
DGUOK	Santa Cruz	Cat#SC-398093
Oxidative phosphorylation cocktail	Abcam	Cat#Ab110413
PGC1 alpha	Abcam	Cat#Ab77210
Acetyl lysine	abcam	Cat#Ab190479
HSP90 beta	Abcam	Cat#Ab32568
MT-ATP8	Santa Cruz	Cat#SC-84231
Cytochrome b	Santa Cruz	Cat#SC-11436
ND6	Santa Cruz	Cat#SC-20510
MTCO1	abcam	Cat#Ab14705
SOD2 (Abcam, #13533	abcam	Cat#Ab13533
PINK1	abcam	Cat#Ab23707
HSP90β	Abcam	Cat#Ab32568
Goat anti-Mouse IgG	ThermoFisher Scientific	Cat#31430
Goat Anti-Rabbit IgG	ThermoFisher Scientific	Cat#656120
Donkey Anti-Goat IgG	ThermoFisher Scientific	Cat#11058
Bacterial Strains		
pzbFGF BL21 Star *E. coli*	Ludwig et al., 2006	N/A
Chemicals, Peptides, and Recombinant Proteins		
B-27® Supplement (50X), serum free	ThermoFisher Scientific	Cat#17504044
B-27® Supplement, minus insulin	ThermoFisher Scientific	Cat#A1895601
Activin A Recombinant Human Protein	ThermoFisher Scientific	Cat#PHC9563
FGF-Basic (AA 10-155) Recombinant Human Protein	ThermoFisher Scientific	Cat#PHG0023
Purified recombinant zebrafish FGF-Basic	Ludwig et al., 2006	Cat#N/A
BMP4 Recombinant Human Protein	ThermoFisher Scientific	Cat#PHC9533
HGF Recombinant Human Protein	ThermoFisher Scientific	Cat#PHG0321
Oncostatin M Recombinant Human Protein	ThermoFisher Scientific	Cat#PHC5015
NADIDE	MicroSource Discovery Systems, Inc.	Spectrum Collection; CAS 53-84-9
Niagen	Chromadex	Cat#ASB-00014332
dAMP	Santa Cruz biotechnology	Cat#Sc-338742
dGMP	Santa Cruz biotechnology	Cat#Sc-225815
SYBR green I nucleic acid gel stain	ThermoFisher Scientific	Cat#S7563
SR 18292	Cayman Chemical	Cat#2095432-55-4
Critical Commercial Assays		
RNeasy mini Kit	QIAGEN	Cat#74106
TURBO DNA-free™ Kit	ThermoFisher/Ambion	Cat#AM1907
M-MLV Reverse Transcriptase	ThermoFisher/Invitrogen	Cat#28025-013
TaqMan^®^ Gene Expression	ThermoFisher/Applied Biosystems	Cat#4369016
Power SYBR Green	ThermoFisher/Applied Biosystems	Cat#4367659
Lactate colorimetric/fluorometric assay kit	Biovision	Cat#607
NAD/NADH colorimetric kit	Biovision	Cat#K337
Pierce BCA protein assay kit	Thermo Fisher	Cat#23227
TMRE mitochondrial membrane potential assay kit	Abcam	Cat#Ab113852
Mitochondrial isolation kit from tissue	Abcam	Cat#Ab110168
Electron transport chain complex I enzyme activity microplate assay kit	Abcam	Cat#Ab109721
Electron transport chain complex II enzyme activity microplate assay kit	Abcam	Cat#Ab109908
Electron transport chain complex III enzyme activity microplate assay kit	Biovision	Cat#K520
Electron transport chain complex IV rodent enzyme activity microplate assay kit	Abcam	Cat#Ab109911
ATP synthase enzyme activity microplate assay kit	Abcam	Cat#Ab109714
QuickExtract™ DNA extraction solution	Epicenter	Cat#QE09050
Experimental Models: Cell Lines		
iPSC-SV20	Yang et al., 2015	N/A
Experimental Models: Organisms/Strains		
DGUOK−/− Rat	Bennett et al., 2016	N/A
Recombinant DNA		
PX459 pSPCas9(BB)-2A-Puro vector	Ran et al., 2013	Addgene Plasmid #62988
DGUOK-pTETon-MCS	This paper	N/A
Oligonucleotides		
DGUOK exon 4 CRISPR guide sequence: catcgagtggcatatctatc	Integrated DNA Technologies	N/A
Forward PCR primer for DGUOK INDELs: atcccacttccaaccaggtatatctttgc	Integrated DNA Technologies	N/A
Reverse PCR primer for DGUOK INDELs: ctggggagaagcctggaggtagatgaag	Integrated DNA Technologies	N/A
RRM2B exon 2 CRISPR guide sequence: tcctaagaaagagttctcgc	Integrated DNA Technologies	N/A
Forward PCR primer for RRM2B INDELs: acaggctctcaaaccaatgc	Integrated DNA Technologies	N/A
Reverse PCR primer for RRM2B INDELs: tctcagtaattccaacacttatcttc	Integrated DNA Technologies	N/A
Primers for qPCR, see Table S2	Integrated DNA Technologies	N/A
Software and Algorithms		
Image Lab software	Bio-Rad	Cat#1709691
GraphPad Prism v6	Graphpad Software, Inc	N/A
DNA-Chip Analyzer (dChip)	Li and Wong, 2001	www.dchip.org
PANTHER classification System	Mi et al., 2013	www.pantherdb.org
STITCH database	Szklarczyk et al., 2016	stitch.embl.de
Other		
Spectrum Collection	Microsource Discovery Systems Inc	www.msdiscovery.com/spectrum.html
HCM BulletKit (CC-3199 & CC-4182)	Lonza	Cat#CC-3198
Seahorse XF96 Cell mito Stress Test system	Agilent	Cat#103015-100

### CONTACT FOR REAGENT AND RESOURCE SHARING

Information and requests for resources and reagents should be directed to and will be fulfilled by the Lead Contact, Stephen A. Duncan (duncanst@musc.edu).

### EXPERIMENTAL MODEL AND SUBJECT DETAILS

#### Cell lines

Human SV20 iPSCs were generated from peripheral blood and characterized previously (Yang et al., 2015). Human wild-type SV20 or DGUOK^Δ14/Δ5^ iPSCs were cultured in mTeSR medium (Ludwig et al., 2006) with 4 ng/ml zbFGF on an E-cadherin-IgG Fc fusion protein matrix (Nagaoka et al., 2010) in 4% O_2_/5% CO_2_ at 37°C with daily medium changes.

#### Animals

The development of the DGUOK deficient rat has been previously described (Bennett et al., 2016) and is maintained at the Medical College of Wisconsin in a specific pathogen-free facility as a homozygous breeding colony, fed Teklad #7034 low-salt chow, under a 14/10-hr light/dark cycle. 8-week-old, 22^nd^ intercross generation, naive DGUOK homozygous deficient rats (167-272-g body weight) were randomly assigned to study groups, group housed, and orally administrated with water (vehicle) (n = 6; 5 males, 1 female) or NR (Niagen, ChromaDex, CA) (n = 7; 3 males, 4 females, 500mg/kg/day) for seven days. Immune competency is unknown in this model. On day 8 the animals were euthanized and liver samples were collected for ATP and electron transport chain activity assays. To prevent circadian variations, all treatments and sample collections were conducted at the same time of the day. All studies were performed in accordance with the Guide for the Care and Use of Laboratory Animals and approved by the MCW Institutional Animal Care and Use Committee.

### METHOD DETAILS

#### Differentiation of Human iPS Cells

SV20 cells were seeded on Matrigel (2 mg/ml)-coated tissue culture plates 24 hours prior to differentiation. Cells were induced to form hepatocyte-like cells as described in a stepwise protocol published previously. {Mallanna and Duncan, 2013, Curr Protoc Stem Cell Biol, 26, Unit 1G.4.}. Specifically, iPSCs were treated with bone morphogenetic protein 4 (BMP4,10ng/ml), fibroblast growth factor 2 (FGF2 20ng/ml), and Activin A(100ng/ml) for 2 days and Activin A(100ng/ml) alone for another 3 days to form endoderm, followed by 5 days of BMP4 (20ng/ml) and FGF2 (10ng/ml) to induce hepatic progenitor cell formation. The hepatic progenitors were then culture with hepatocyte growth factor (HGF 20ng/ml, day 10 to day 15) and Oncostatin M (OSM 20ng/ml, day 15 to day 20) to generate hepatocyte-like cells.

#### ATP, lactate, and NAD^+^ Measurements

ATP levels in cultured cells were measured using CellTiter-Glo assay (Promega, #G7570) and Tissue ATP levels were measured using an ATP assay kit (Abcam, #ab83355), according to the manufacturer’s instructions. Lactate levels were measured using lactate colorimetric/Fluorometric assay Kit (Biovision, #607) and NAD^+^ levels were measured using NAD/NADH quantitation colorimetric kit (Biovision, #K337) following manufacturer’s direction. Results were normalized to protein content determined by the Pierce BCA protein assay kit (Thermo Fisher, #23227).

#### Drug screening

Human DGUOK^Δ14/Δ5^ iPSCs were seeded on thirty 96-well plates and induced to differentiate until day 15. Drugs from the Spectrum collection (MicroSource, CT) were individually applied between day 15 to day 20 of differentiation. In each 96-well plate, 8 wells were untreated, 8 wells were treated with DMSO, and the remaining wells were treated with drugs at a concentration of 5 μM. At the end of day 20, cells were treated with 20mM 2-DG (Sigma-Aldrich, #D8375) for 24hr and a luminescent assay (CellTiter-Glo (Promega, #G7570)) was performed to detect ATP levels. Z scores were calculated based on ATP levels to identify hits. Bioinformatics analyses of the hits were performed using STITCH (Szklarczyk et al., 2016) and the PANTHER classification system (Mi et al., 2013).

#### Measurement of OCR and ECAR

iPSCs were plated on Matrigel (2mg/ml)-coated Agilent Seahorse XF96 Microplates (Agilent, #101085-004) and differentiated to hepatocyte-like cells. Seahorse XF96 Analyzer (Agilent, CA) was used to measure the basal levels of OCR and ECAR, as well as the OCR and ECAR of cells in the presence of electron transport chain inhibitors and uncouplers (oligomycin, 1 μM; carbonyl cyanide-p-trifluoromethoxyphenylhydrazone, 1 μM; rotenone, 2 μM; antimycin A, 1 μM). A BCA assay was performed on tested wells and OCR and ECAR results were normalized to protein content. Statistics and graphs were generated using Agilent Seahorse Wave Desktop software.

#### Measurement of MMP

TMRE-Mitochondrial membrane potential assay kit (Abcam, #ab113852) was used to measure MMP. Live hepatocyte-like cells were first incubated with culture medium containing 200nM TMRE for 30 minutes at 37 degree, then rinsed three times with PBS and incubated with culture medium containing 50 nM TMRE. Cells were imaged by Zeiss LSM 880 laser scanning microscopy or ZOE fluorescent cell imager (Biorad, CA). For quantitative assays, we measured TMRE staining using Synergy HTX microplate reader (BioTek, VT) following manufacturer’s instruction.

#### Measurement of electron transport chain activities

Mitochondria were isolated from the DGUOK deficient rat tissues using mitochondria isolation kit for tissue (Abcam, #ab110168). The activities of electron transport chain complex I, II, III, IV, V were determined using complex I enzyme activity microplate assay kit (Abcam, #ab109721), complex II enzyme activity microplate assay kit (Abcam, #ab109908), mitochondria complex III activity assay kit (Biovision, #K520), complex IV rodent enzyme activity microplate assay kit (Abcam, #ab109911), and ATP synthase enzyme activity microplate assay kit (Abcam, #ab109714), respectively, following manufacturer’s instruction.

#### Immunostaining

Cultured cells were fixed with 4% PFAfor30 minutes and made permeable using 0.5% Triton X-100 in PBS for 15 minutes. Cells were treated with 3% BSA in PBS for 30 minutes followed by overnight incubation with primary antibody at 4°C. Antibodies used were HNF4A (Santa Cruz, #sc-1556, 1:250), Albumin (Cedarlane, #CL2513A, 1:500), and AFP (Sigma, #A8452, 1:250). Cells were rinsed with PBS 3 × 5 minutes and incubated with DAPI (1 μg/ml) and secondary antibody for 1 hour at room temperature. Alexa fluor antibodies (594 nm anti-goat, 488 nm anti-rabbit, 488 nm anti-goat) were used at 1:1000 dilution. Images were processed using Adobe Photoshop to optimize brightness/contrast. Control and experiment wells were processed identically.

#### Immunoblot and Immunoprecipitation

Whole cell lysates were collected using NP-40 or RIPA buffer with protease inhibitor cocktail (ThermoFisher Scientific, NY, #78443). 30mg total protein was separated by SDS–PAGE using Any kD™ Mini-protean TGX stain-free™ precast gels (BioRad, CA, #4568123), and transferred to PVDF membranes using the Trans-Blot Turbo™ Transfer System (BioRad, CA, #1704155). Membranes were incubated overnight with antibodies against DGUOK (Santa Cruz, #sc-398093, 1:200), HSP90β (Abcam, #ab32568, 1:100000), MT-ATP8 (Santa Cruz, #sc-84231, 1:200), cytochrome b (Santa Cruz, #sc-11436, 1:200), ND6 (Santa Cruz, #sc-20510, 1:200), MTCO1 (abcam, #ab14705, 1:1000), oxidative phosphorylation antibody cocktail (UQCRC2, ATP5A, MTCO1) (Abcam, #ab110413, 1:250), PGC1 alpha (Abcam, #ab77210, 1:400), SOD2 (Abcam, #13533, 1:5000), PINK1 (Abcam, #23707, 1:500) or Acetyl lysine (Abcam, #ab190479, 1:500) at 4°C. HRP-conjugated secondary antibodies were used at a dilution of 1:2000. Protein levels were calculated using BioRad stain-free Imaging System and were normalized to total protein using Image Lab software from BioRad. Immunoprecipitation was performed using Catch and Release^®^ V2.0 kit (EMD Millipore, CA, #17-500) following the manufacturer’s directions.

#### Quantitative Real-Time PCR analysis

RNA was isolated from SV20 cells or iPSC-derived hepatocyte-like cells using the RNeasy mini Kit (QIAGEN, #74106). Genomic DNA was removed using the TURBO DNA-free™ Kit (ThermoFisher/Ambion, NY, #AM1907). First strand cDNA was synthesized using M-MLV Reverse Transcriptase (ThermoFisher/Invitrogen, NY, #28025-013). Quantitative real-time PCR was performed on a BioRad CFX384 real-time PCR machine using TaqMan^®^ Gene Expression assay (ThermoFisher/Applied Biosystems, NY, #4369016) or Power SYBR Green PCR assays (ThermoFisher/Applied Biosystems, NY, #4367659) following the manufacturer’s directions. SYBR green primers and TaqMan assays are listed in Table S2.

#### Measurement of mtDNA content

mtDNA staining was performed on Day 20 iPSC-derived hepatocyte-like cells. Cells were incubated with culture medium containing SYBR green I Nucleic Acid Gel Stain (1:100,000 dilution) and imaged using Zeiss LSM 880 laser scanning microscopy. mtDNA copy number were measured by SYBR green qPCR using primers targeting mitochondrial tRNA leucin and nuclear gene beta-2-microglobin (B2M). The relative mtDNA levels were calculated based on the ΔCq value between mitochondrial and nuclear genes (Bai and Wong, 2005). Primers used are listed in Table S2.

#### Electron Microscopy

D20 hepatocytes were washed with Sorensen’s buffer and fixed with 1% glutaraldehyde in Sorensen’s buffer for 30 min. Cells were collected by scraping followed by centrifugation at 800 × g for 7 min and fresh fixative was added. Samples were then processed for conventional electron microscopy and embedded in Embed 810 resin (Electron Microscopy Sciences, Hatfield, PA). Thin sections were examined on a JEOL JEM-1010 microscope.

#### CRISPR/Cas9 Genome Editing

CRISPR guide RNAs targeting exon 4 of the *DGUOK* or exon 2 of *RRM2B* were designed following the protocol established by Zhang and colleagues (Ran et al., 2013). A guide sequence (catcgagtggcatatctatc) was cloned into PX459 pSPCas9(BB)-2A-Puro vector (Ran et al., 2013). The plasmid was introduced into SV20 cells by electroporation using a BTX electroporator. Electroporated iPSCs were cultured on Matrigel for 24 hours in the presence of ROCK inhibitor Y27632 (StemRD, CA, #146986-50-7) and then treated with 1 μg/ml Puromycin for two days. Cells that survived the selection were expanded until clones could be collected. Genomic DNA was extracted from the clones using QuickExtract™ DNA extraction solution (Epicenter, WI, #QE09050). The targeted regions of were amplified using Herculase Fusion Polymerase (Agilent, CA, #600675) and run on Novex^®^ 4%–20% TBE gels (ThermoFisher/Invitrogen, CA, #EC6225BOX) to detect INDELs *(DGUOK* For: atcccacttccaaccaggtatatctttgc, Rev: ctggggagaagcctggaggtagatgaag; *RRM2B* For: acaggctctcaaaccaatgc, Rev: tctcagtaattccaacacttatcttc). Amplicons were cloned into plasmid and subjected to nucleotide sequencing to confirm the identity of the INDELs.

### QUANTIFICATION AND STATISTICAL ANALYSIS

In the drug screen, Z-factor was defined based on the means and standard deviations of both the positive and negative control values and calculated using an online Z-factor calculator (http://www.screeningunit-fmp.net/tools/z-prime.php).

Primary hits were identified by determining the z-score for each drug calculated plate by plate. Drugs with a z score ≥ 3 were considered as primary hits. All the primary hits were then repeated in triplicate, and the P values were calculated using Student’s t test (unpaired). To determine the significance of the difference between control and experimental results generated by ATP assay, NAD measurement, seahorse analysis, lactate assay, mtDNA content measurement, immunostaining pixel intensity measurement (TMRE), immunoblot densitometry, and RT-qPCR assays, statistical significance was determined using unpaired Student’s t test. Graphs were generated using Excel, and statistics were calculated with GraphPad Prism. Statistic parameters in each experiment (value of n, SEM, orSD) are reported in the figure legends. In the animal experiments, DGUOK-deficient rats were randomly assigned to control (n = 6) and experimental group (n = 7) to test the efficacy of NR. Unpaired Student’s t test was performed to determine the statistical significance in the mitochondrial activity assays.

## Supplementary Material

1

2

3

## Highlights

- DGUOK-deficient iPSCs are generated using CRISPR/Cas9
- DGUOK-deficient iPSCs give rise to hepatocytes that recapitulate MTDPS3
- A drug screen using iPSC-derived hepatocytes reveals potential treatments for MTDPS3
- NAD improves mitochondrial function in MTDPS3 without rescuing mtDNA depletion
