# Supplementary material for: A Screen Using iPSC-Derived Hepatocytes Reveals NAD+ as a Potential Treatment for mtDNA Depletion Syndrome
Source: Cell Rep. Author manuscript; Available in PMC 2018 Dec 11. (PMC6289059; doi:10.1016/j.celrep.2018.10.036)
Supplement: 1 [file NIHMS1512154-supplement-1.pdf]

Cell Reports, Volume *25*

Supplemental Information

A Screen Using iPSC-Derived Hepatocytes Reveals NAD$^+$ as a Potential Treatment for mtDNA Depletion Syndrome

Ran Jing, James L. Corbett, Jun Cai, Gyda C. Beeson, Craig C. Beeson, Sherine S. Chan, David P. Dimmock, Lynn Lazcares, Aron M. Geurts, John J. Lemasters, and Stephen A. Duncan

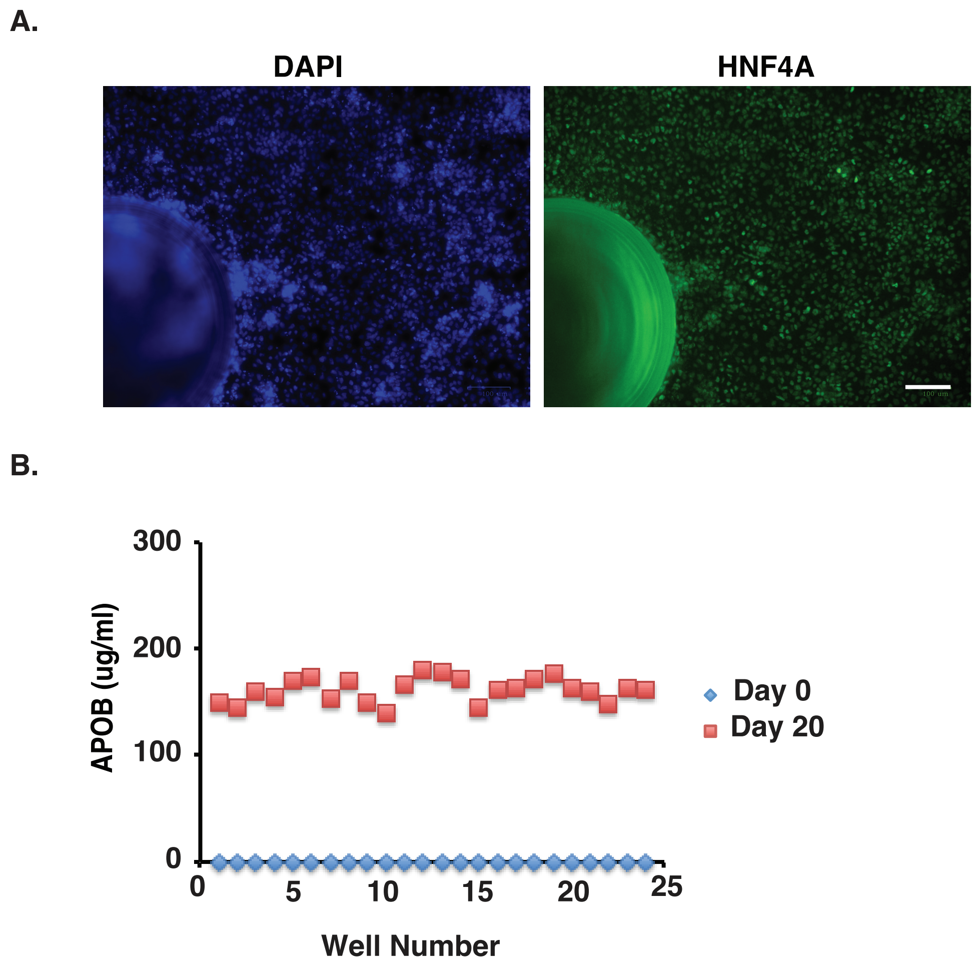

Fig. S1. Related to Figure 1. Expression of hepatocyte markers during the differentiation of iPCSs in 96–well Seahorse plates. A) Immunostaining to detect HNF4a after 20 days of differentiaiton of iPSCs. Cells were counterstained with DAPI to identify nuclei. Scale bar = 100μM. B) Levels of APOB were measured in the medium of iPSCs (blue) or in iPSC-derived hepatocytes (day20; red) using ELISA on individual wells.

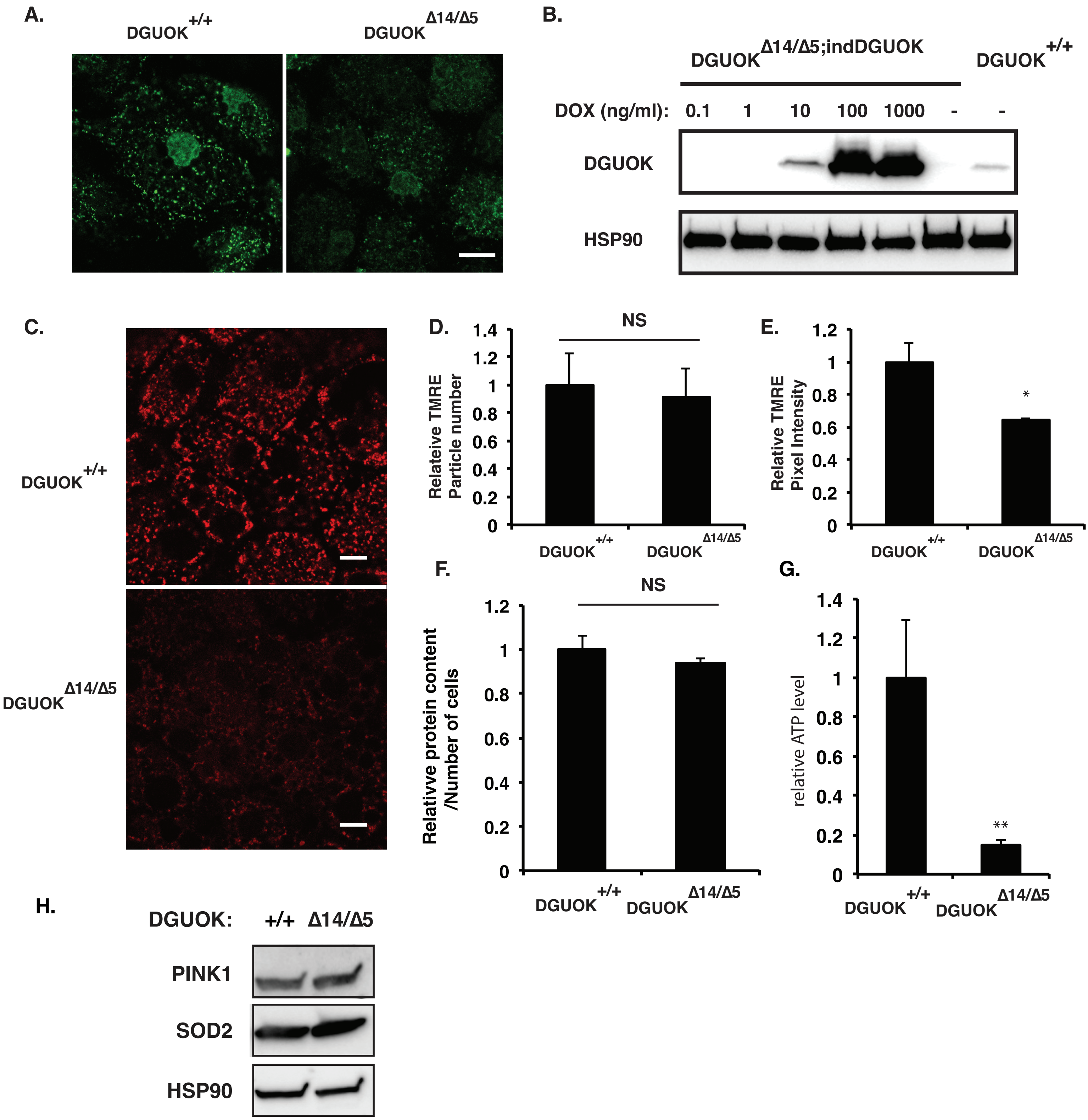

Fig. S2. Related to Figure 3. DGUOK deficiency results in mitochondria dysfunction in iPSC derived hepatocytes
A) SYBR green I (1:100,000 dilution) staining of mtDNA in wildtype and DGUOK$^{\Delta14/\Delta5}$ iPSC-derived hepatocytes (day 20). Scale bar=10μM. B) Immunoblot showing DGUOK protein levels in DGUOK$^{\Delta14/\Delta5,indDGUOK}$ cells treated with different concentrations of Doxycyline (DOX). HSP90 was used as a loading control. C)TMRE staining in wildtype and DGUOK$^{\Delta14/\Delta5}$ iPSC derived hepatocytes. Scale bar=10μm. D - G) Bar charts showing particle number (D) and pixel intensity (E) of TMRE staining, protein content (F) per cell of control and DGUOK deficient hepatocytes normalized to cell number, and (G) cellular ATP content in control and DGUOK deficient hepatocytes cultured in galactose for 24 hrs, N=4 biological replicates; * p = ≤0.05, ** p≤0.01. H) Immunoblot detecting the level of a characteristic mitophagy marker (PINK1) and mitochondrial ROS scavenger (SOD2) in control and DGUOK-deficient hepatocytes. HSP90 was used as a loading control.

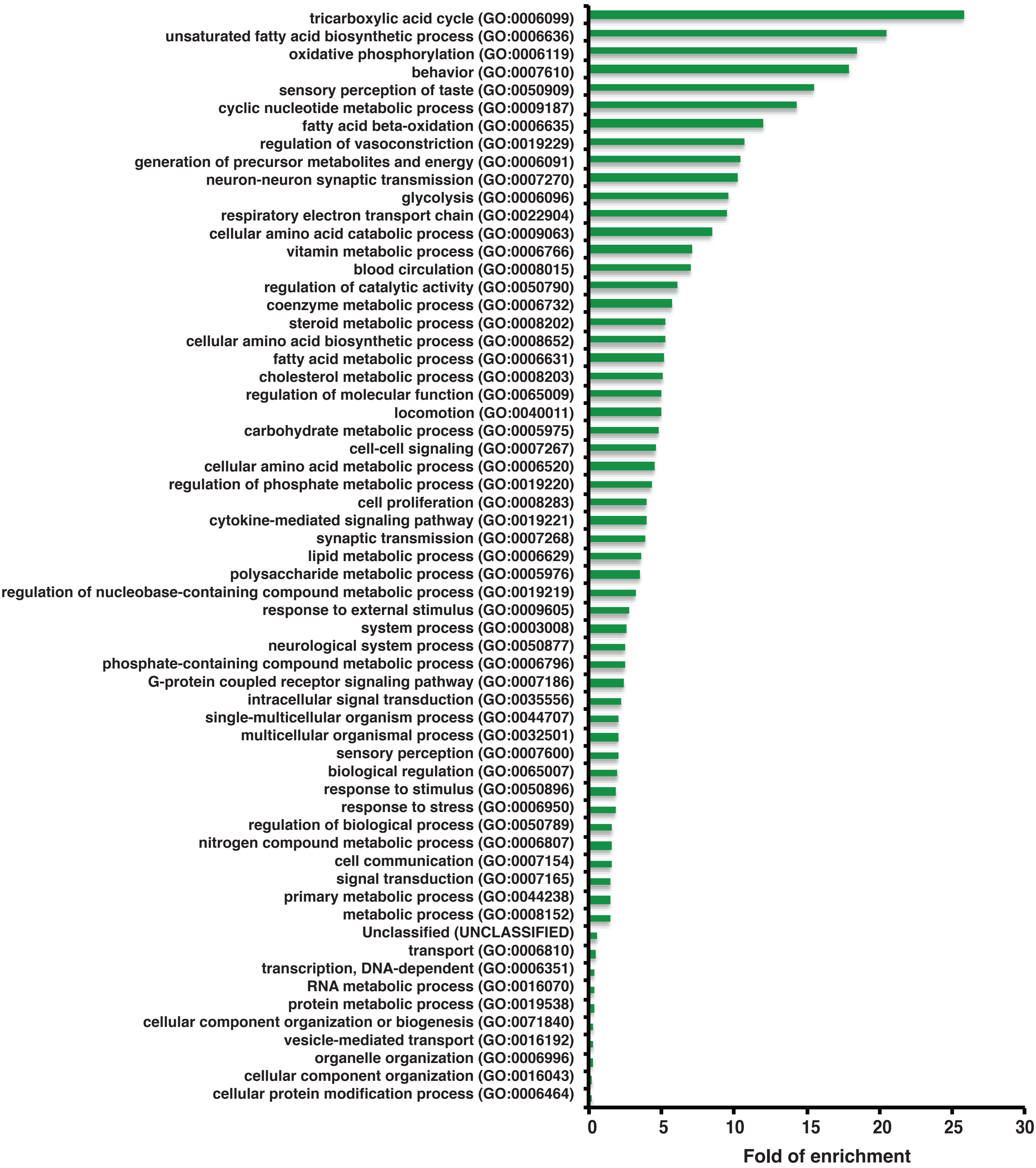

Fig. S3. Related to Figure 4. GO analysis of proteins targeted by the 34 drugs found to increase ATP production in DGUOK deficient iPSC-derived hepatocyte-like cells. Bar graph showing the fold over representation of biologcial processes invovling proteins targeted by the drugs.

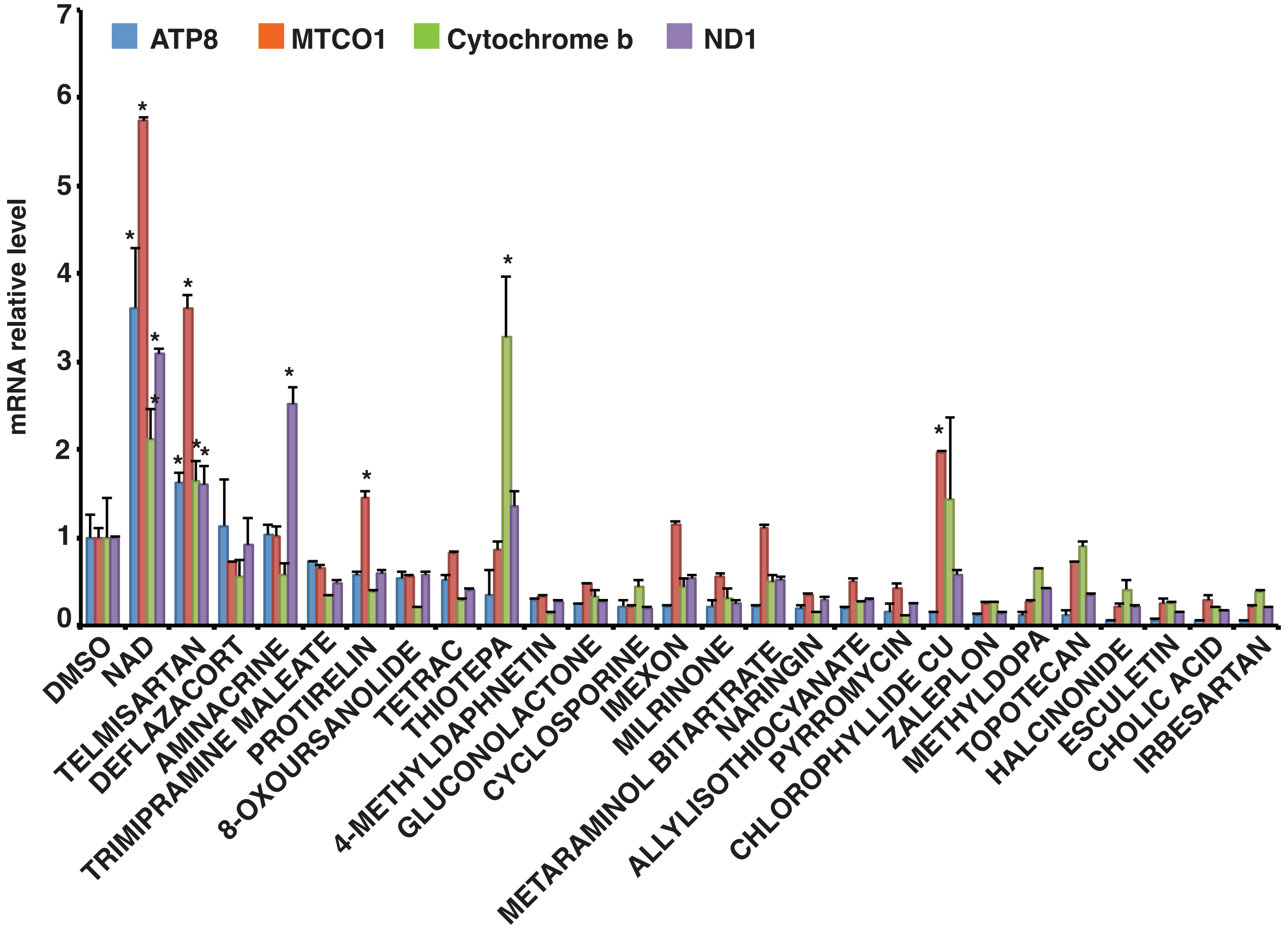

Fig. S4. Related to Figure 4. Expression of ETC genes in DGUOK deficient iPSC-derived hepatocyte-like cells treated with drugs. Bar graph showing fold change in the steady-state mRNA levels of mitochondrial ETC genes in DGUOK$^{\Delta14/\Delta5}$ iPSC-derived hepatocytes treated with vehicle (DMSO) or hits. (mean±SEM, N=3, *p≤0.05)

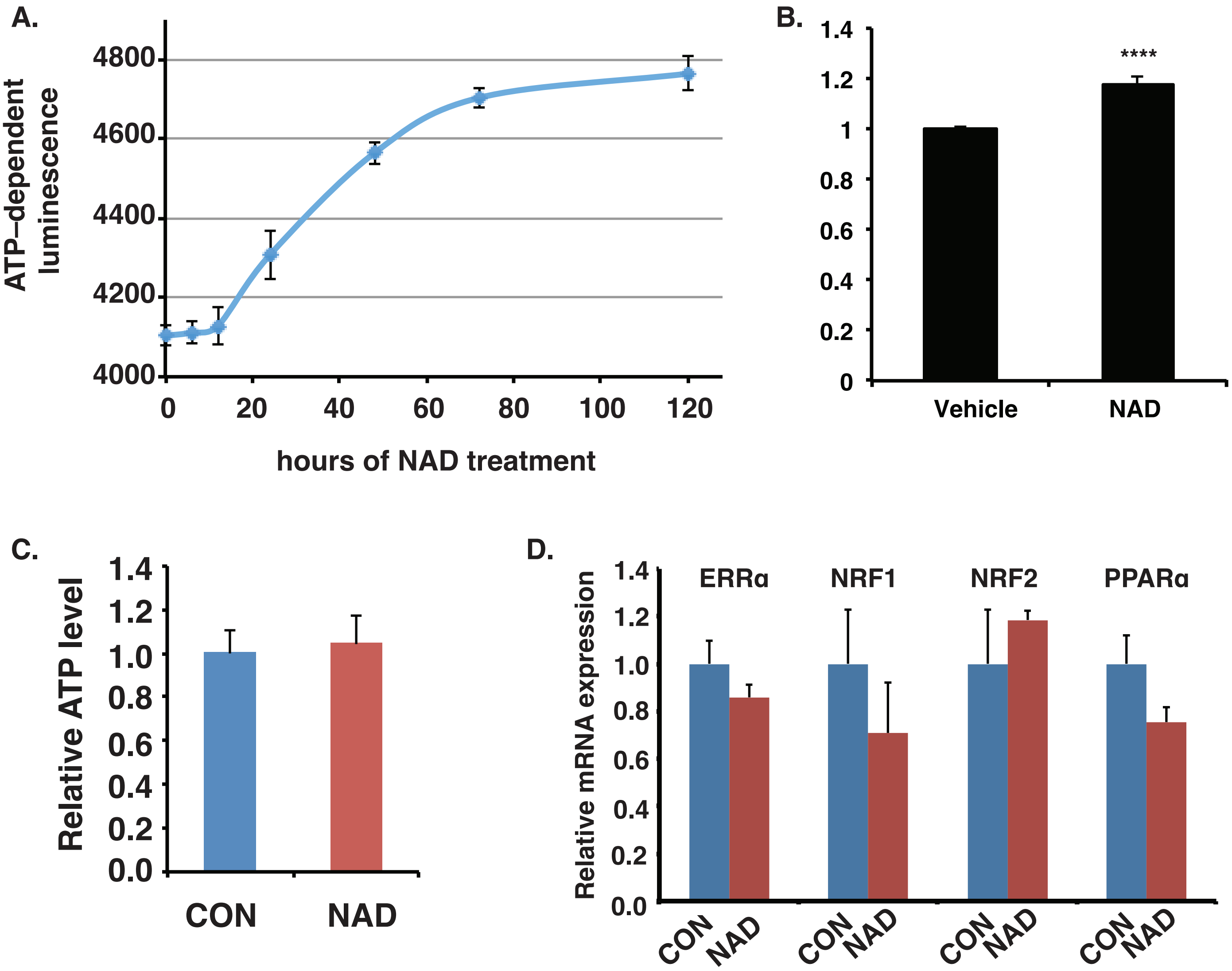

Fig. S5. Related to Figures 5 and 6. Response of hepatocyte-like cells to NAD treatment.
A) Graph showing impact of NAD on ATP production over time. DGUOK-deficient hepatocytes treated with 5μM NAD for 0, 6, 12, 24, 48, 72, and 120 hoursbefore ATP levels were detected by luminescence assay at Day 20. (n=8, mean ± SEM). B) DGUOK-deficient hepatocyte-like cells were treated with vehicle (DMSO) or 5uM NAD for 5 days before measuring ATP levels luminescence assay at day 25. (n=8, mean ± SEM, **** p≤0.0001). C) Bar graph showing ATP levels in control and NAD treated (5uM) wild type iPSC-derived hepatocyte-like cells. B) Bar graph showing relative steady-state mRNA levels of PGC1α targets (ERRα, PPARα, NRF1, and NRF2) in control and NAD treated wild type iPSC-derived hepatocyte-like cells .

Table S2. Related to STAR methods. Primers used for PCR amplifications

Gene	Forward 5'-3'	Probe 5'-3'	Reverse 5'-3'
**TaqMan PCR primers**			
HNF4A	TGG ACA AAG ACA AGA GGA ACC	56-FAM/TCTGGACGG/ZEN/CCTCCTTCTTCATGC/3IABkFQ	ATA GCT TGA CCT TCG AGT GC
RPL13A	GGCCACACTGTTGATGACA	56-FAM/TTGCACAAA/ZEN/GCCTCAACACCTCC/3IABkFQ	CCATAATCCCCAGCAATCTCA
CYP3A4	ACCAGTGGAAAACTCAAGGAG	TGATCACATCCATGCTGTAGG	TTGGTGAGAAATCTGAGGCGGGAAG
ASGR1	TCCTTTCTGAGCCATTGCC	CGTGAAGCAGTTCGTGTCTGACCT	TGAAGTCGCTAGAGTCCCAG
**SYBR green PCR primers**			
RPL13A	CTCAAGGTGTTTGACGGCATCC		TACTTCCAGCCAACCTCGTGAG
APOB	AGAGGACAGAGCCTTGGTGGAT		CTGGACAAGGTCATACTCTGCC
HSP90	GGATGACAGCGGTAAGGATAAG		GAGCCCGACGAGGAATAAATAG
MCAD	ACAGGGGTTCAGACTGCTATT		TCCTCCGTTGGTTATCCACAT
VLCAD	TCAGAGCATCGGTTTCAAAGG		AGGGCTCGGTTAGACAGAAAG
ACOX1	GAGGTCCACGAATCTTACAAGCA		TTGCACACAGGCGCTTTCT
CPT1A	TCCAGTTGGCTTATCGTGGTG		CTAACGAGGGGTCGATCTTGG
OGDH	AGATCATCCGTCGGCTGGAGATGG		CTTCTCAGAGGACCACTTCCGCTG
CS	CAACTCAGGACGGGTTGTTCCAGG		GTAGTAATTCATCTCCGTCATGCC
IDH3A	ACATCCTTAGTGACTTGTGTGCAG		GCATTGCCTCCCAAATCTTTTGTC
IDH3B	GATGTGCTTGTGATGCCCAATCTC		GTGATACTCAAGATTAAGATGCCG
NRF1	AGCAAAAGCAGAGGGTTTCA		CTGTGTTTGCGTTTGCTGAT
NRF2	GAGAGCCCAGTCTTCATTGC		TGCTCAATGTCCTGTTGCAT

PPARα	CAGAACAAGGAGGCGGAGGTC		TTCAGGTCCAAGTTTGCGAAGC
ERRα	CCTCTGTGACCTCTTTGACC		TACTGACATCTGGTCAGACA
TFAM	CCGAGGTGGTTTTCATCTGT		GCATCTGGGTTCTGAGCTTT
TFB1M	ATGGCTCAGTACCTCTGCAATG		TGGGCTGTATCAAGGGAGTGA
TFB2M	ATCCCGGAAATCCAGACTTGT		GACCAAGGCTCCATGTGCA
ND1	ATGGCCAACCTCCTACTCCTCATT		TTATGGCGTCAGCGAAGGGTTGTA
COX1	ACCCTAGACCAAACCTACGCCAAA		TAGGCCGAGAAAGTGTTGTGGGAA
CYTB	AGTCCCACCCTCACACGATTCTTT		AGTAAGCCGAGGGCGTCTTTGATT
ATP8	ACCGTATGGCCCACCATAATTACC		TTTATGGGCTTTGGTGAGGGAGGT
mt-tRNA-Leu	CACCCAAGAACAGGGTTTGT		TGGCCATGGGTATGTTGTTA
B2G	TGCTGTCTCCATGTTTGATGTATCT		TCTCTGCTCCCCACCTCTAAGT
